# Advancing NanoLuc Luciferase Stability beyond Directed Evolution and Rational Design through Expert-Guided Deep Learning

**DOI:** 10.1021/acscatal.5c08789

**Published:** 2026-01-27

**Authors:** Spencer Gardiner, Joseph Talley, Tyler Green, Christopher Haynie, Corbyn Kubalek, Matthew Argyle, William Heaps, Joshua Ebbert, Deon Allen, Dallin Chipman, Bradley C Bundy, Dennis Della Corte

**Affiliations:** † Department of Physics and Astronomy, 6756 Brigham Young University, Provo, Utah 84602, United States; ‡ Department of Chemical Engineering, 6756 Brigham Young University, Provo, Utah 84602, United States

**Keywords:** enzyme engineering, luciferase, thermostability, rational design, inverse protein folding, cell-free protein synthesis, deep learning

## Abstract

Engineered luciferases have transformed biological imaging and sensing, yet optimizing NanoLuc luciferase (NLuc) remains challenging due to the inherent stability-activity trade-off and its limited sequence homology with characterized proteins. We report a hybrid approach that synergistically integrates deep learning with structure-guided rational design to develop enhanced NLuc variants that improve thermostability and thereby activity at elevated temperatures. By systematically analyzing libraries of engineered variants, we established that modifications to termini and loops distal from the catalytic center, combined with preservation of allosterically coupled networks, effectively increase thermal resilience while maintaining enzymatic function. Our optimized variants—notably B.07 and B.09—exhibit substantial thermostability enhancements (increased melting temperatures of 7.2 and 5.1 °C, respectively), leading to the sustained activity of a high-activity mutant at elevated temperatures. Molecular dynamics simulations and protein folding studies elucidate how these mutations favorably modulate conformational landscapes without perturbing the substrate binding architecture. Beyond providing a thermostabilized tool for bioluminescence applications, our integrated methodology presents a framework for engineering enzymes when traditional homology-based approaches fail and stability-activity constraints present formidable barriers to improvement.

## Graphical abstract

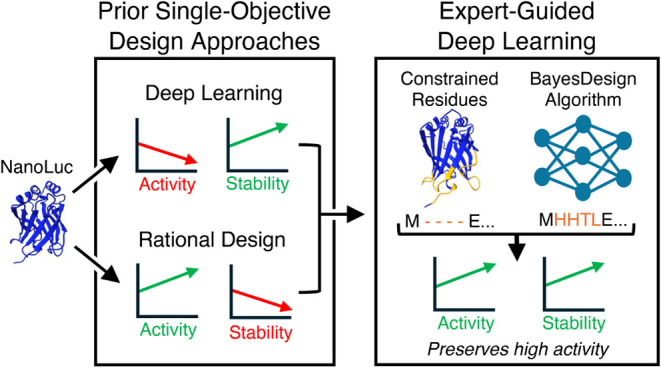

## Introduction

Bioluminescence, the production and emission of cold light by living organisms, provides a distinct optical readout for biological processes, distinguishing it from fluorescence, combustion, or electrical illumination.^1,2^ Luciferases, enzymes responsible for this effect, have become indispensable tools in protein–protein and protein–ligand interaction studies,^3–6^ gene regulation,^6^ protein stability monitoring,^7^ and bioimaging.^8,9^ Among these, bioluminescence resonance energy transfer (BRET)-based sensors have expanded the capabilities of real-time, noninvasive imaging, offering high signal-to-background ratios and detection sensitivity in deep tissues.^7,10,11^

Despite their utility, natural luciferases can suffer from poor folding, low activity, and susceptibility to proteolytic degradation in mammalian cells, limiting their application.^12–14^ Engineering efforts have sought to overcome these challenges by *de novo* design or optimizing existing luciferases for increased brightness, stability, and compatibility with synthetic luciferins that offer desirable photophysical properties and higher substrate specificity.^12^ *De novo* design has successfully created several luciferases that have high thermal stability (*T*_melt_ > 95 °C); however, these enzymes are substantially less luminescent than naturally derived luciferases.^12,15^

While Firefly and Renilla luciferases have been the traditional standards in bioassays and molecular imaging due to their well-characterized properties, the discovery and subsequent optimization of a deep-sea shrimp luciferase yielded the NanoLuc (NLuc) enzyme, a compact (19.1 kDa), stable luciferase with over 150-fold increase in luminescence relative to its natural precursors.^7,16^ Its monomeric nature and unbiased distribution in cells make it a versatile platform for numerous applications, including bioluminescent imaging and biosensing.^7^ Its split variants (NanoBiT) have aided protein interaction studies by providing a dynamic and reliable reporter system.^10,17^ However, activity-enhanced NLuc variants exhibit lower thermal stability than the original NLuc,^18^ limiting their use in higher temperature applications, such as wastewater monitoring,^19^ food processing,^20^ and fermentation control.^21^

Our recent work showed that deep learning–based sequence optimization using BayesDesign can generate NLuc variants with markedly enhanced thermostability.^22^ However, these variants suffered a substantial loss of bioluminescent activity, underscoring a key limitation: stability-focused computational design alone is inadequate for optimizing functional performance in bioluminescent enzymes. Established rational design and deep learning methodologies frequently leverage homology-guided insights to preserve activity.^23–27^ However, NLuc shares limited sequence homology with known proteins,^28^ rendering homology-based design approaches, such as those employed by Sumida et al.,^29^ ineffective for this enzyme. Additionally, the vast number of possible mutations for NLuc (∼10^220^) provides motivation for experts to guide deep learning-based algorithms with guardrails that preserve functional activity by limiting which residues can be mutated. Herein, experts are defined as researchers with extensive understanding of the structure and function of the target protein.

Structural studies have provided critical insights into NLuc architecture and function, including the discovery of a homotropic negative allosteric mechanism.^30^ Rational design approaches based on these insights have successfully enhanced NLuc activity; however, the stability of the most active mutants was not previously reported.^18^

Here, we demonstrate that integrating rational design with deep learning can overcome the stability-activity trade-off and develop superior NLuc variants with enhanced stability, leading to increased activity at elevated temperatures. In this hybrid approach, structural and mechanistic insights are used to identify potentially mutable regions of the protein, which are then redesigned using deep learning tools. We report how this initial application enabled the discovery of novel NLuc variants with improved thermal stability that could expand their operational utility in bioimaging, biosensing, and other bioluminescence-based applications.

## Results and Discussion

### Screening Campaign

Recent efforts to optimize NLuc activity^18^ demonstrated that rational design can go beyond directed evolution, although we found the melting temperature to decrease by 8.6 °C (as will be described later). Conversely, our previous work^22^ employed a deep learning approach to engineer highly stable NLuc variants, though these lacked detectable enzymatic activity. In the present study, we addressed these complementary limitations by developing an integrated approach that synergistically combines rational design principles with deep learning, which will hereafter be referred to as expert-guided deep learning. Specifically, this approach uses rational design to define permissible regions within protein sequence space, which were subsequently subjected to optimization by the inverse-folding model BayesDesign, resulting in the development of two libraries of NLuc variants (A and B). This hybrid methodology effectively harnesses the strengths of both approaches while mitigating their individual shortcomings. A schematic comparison of these methodological progressions is presented in Figure 1.

**Figure 1:**
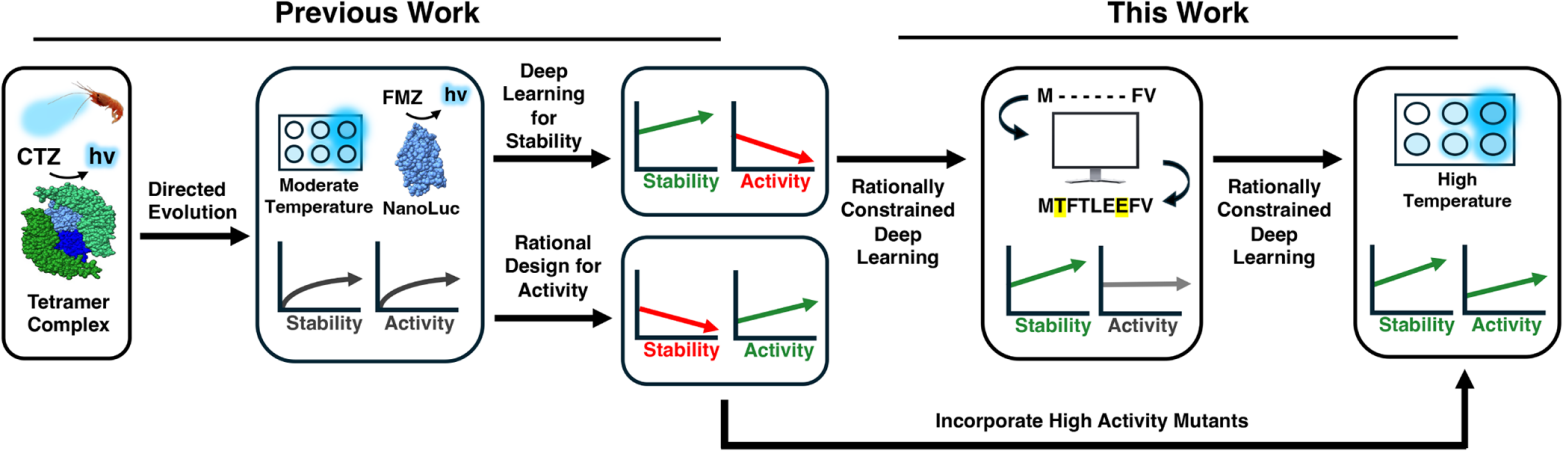
NanoLuc originated from deep-sea shrimp via directed evolution. Previous efforts improved either stability at the cost of lower activity using deep learning or improved the activity through rational design at the cost of reduced thermal stability. In this study, we apply expert-guided deep learning principles to rationally constrain the BayesDesign algorithm to improve the thermal stability of the original NanoLuc and the activity-enhanced NanoLuc while maintaining their original activity through two iterative rounds of computational prediction and experimental validation.

Figure 2 presents an in-depth overview of five sequential rounds of NLuc engineering using the BayesDesign deep learning algorithm and subsequent experimental validation. While we previously reported the experimental thermostability—measured as percent solubility after heat treatment—for selected round 2 variants, we now provide complete data across all rounds for methodological comparison.^22^ Initial rounds (1–3) primarily constrained the number of sites the deep learning model could mutate. Round 1 (1.01–1.10) was the least restrictive, requiring only 29–36% homology with the original wild-type NLuc sequence. Round 2 was restricted to 48–96% homology, and round 3 mutants were constrained to at least 99% homology. Variants in rounds 2 and 3 showed markedly improved solubility at elevated temperatures, even when restricted to only two or three mutations. However, activity was consistently lost and typically undetectable. To overcome this limitation, our current approach combines expert-guided rational design with BayesDesign to identify key mutable regions and generate libraries A and B.

**Figure 2:**
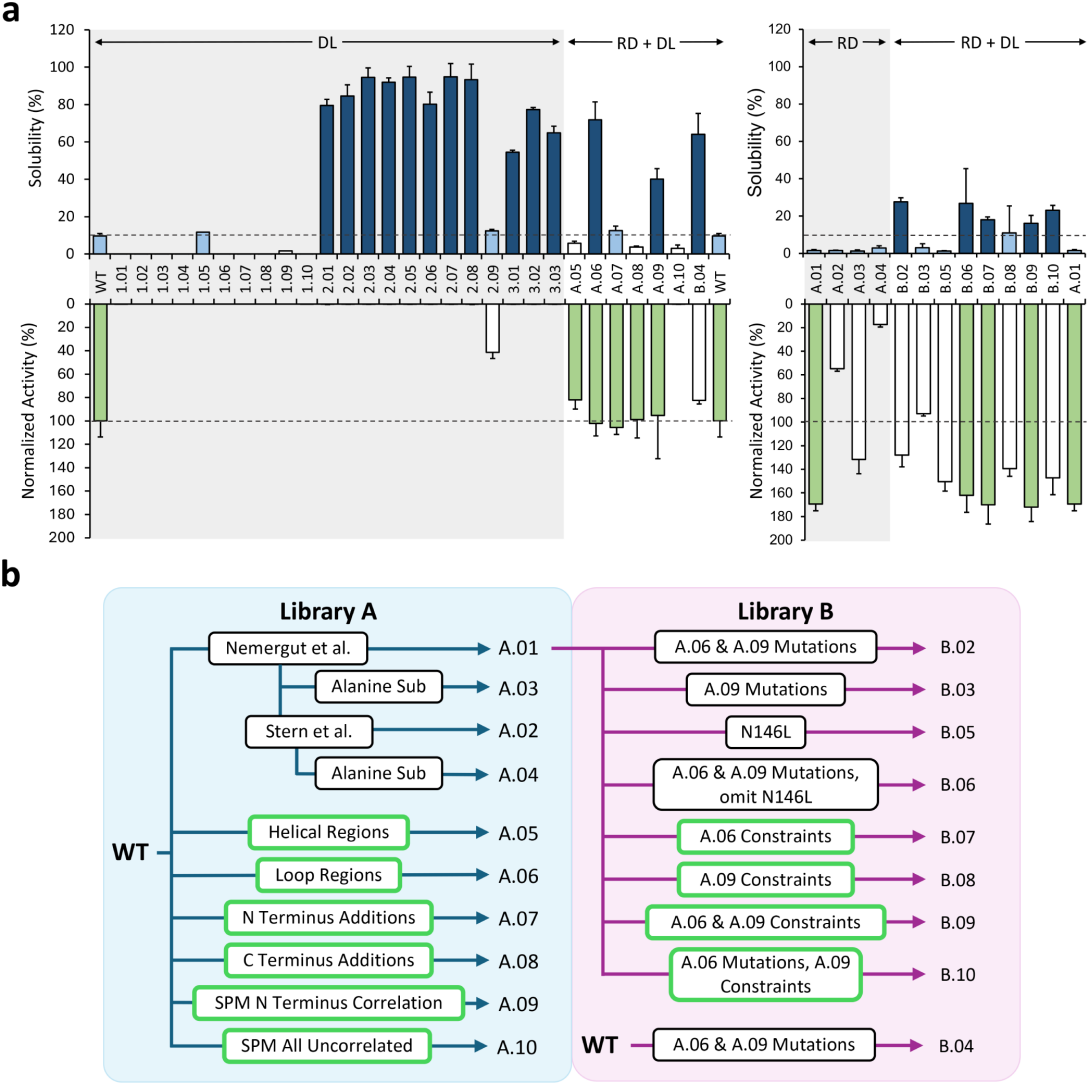
a) Comprehensive solubility and activity results from five sequential rounds of NanoLuc variants. Left chart presents variants derived from the wild-type sequence, while right chart presents variants derived from the A.01 activity-enhanced sequence, with parent sequences shown on both sides of the charts for facile comparison. Solubility was measured at 57 °C (Rounds 1–3) or 60 °C (libraries A and B). Activity normalized to wild-type was measured at 30 °C (Rounds 1–3, library A) or 37 °C (library B). Dashed gray lines indicate baseline wild-type activity and solubility thresholds. Bar color indicates performance when compared to the parent sequence, with white bars indicating lower performance, moderate shading indicating similar performance, and dark shading indicating greater performance. b) A schematic depicting the lineage (arrows from starting sequences) and design strategies (box texts) of variants in libraries A and B. Green borders represent sequences that were generated using BayesDesign. SPM refers to the Shortest Path Method.

Library A consists of four sequences derived from work by Nemergut et al.^18^ and Stern et al.,^22^ and six sequences generated using a combination of rational design and BayesDesign. Variant A.01, a sequence previously characterized as exhibiting enhanced activity with the substrate furimazine (FMZ) by Nemergut et al.,^18^ was found to be highly active (169.4 ± 5.8% of wild-type, Figure 2a) but with compromised thermostability (1.5 ± 0.5% of postheat-treatment solubility). A.02 incorporates a stabilizing mutation identified in Round 3 (V40T) into the A.01 sequence, while A.03 and A.04 are alanine substitutions of residues 91 and 40, respectively. Variants 5–10 were generated by mutating the original “wild-type” NLuc sequence with BayesDesign, constrained by a variety of masks. Each mask specifies which residue positions can mutate and was created using a unique rational design approach: only helix regions (A.05), only loop regions (A.06), C-terminus extension of 5 residues (A.07), N-terminus extension of 5 residues (A.08), only areas dynamically correlated with the N-terminus as determined by shortest path method (SPM) analysis (A.09), or residues with minimal dynamic correlation as determined by SPM analysis (A.10).^31^ Variant A.06 achieved remarkable stability enhancement (71.8 ± 9.6% solubility compared to 9.7 ± 1.2% for wild-type postheat-treatment) while maintaining robust enzymatic activity (102.0 ± 10.9%). Similarly, variant A.09 demonstrated substantial stability improvement (40.1 ± 5.6% solubility) while preserving 95.4 ± 36.9% of wild-type activity. The alanine substitution at R91 (A.03) confirmed the importance of A.01 mutations for enhanced activity. The mutation in A.03 did not affect solubility but did decrease activity relative to A.01. Terminal extension variants showed that C-terminal modifications (A.07) modestly improved both stability and maintained activity, while N-terminal additions (A.08) decreased stability. Mutating residues with minimal dynamic correlation significantly decreased activity (A.10). Comprehensive numerical values for all variants’ activities and solubilities are provided in Table S1.

In library B, we systematically recombined mutations from the best-performing library A variants, with all variants except B.04 using A.01 as the template sequence. B.04 was designed as a combination of A.06 and A.09 mutations. B.04 showed stability and activity below A.06, indicating that the A.06 and A.09 mutations are not cooperative. Variants B.02–03 and B.05–06 were combinations of activity-enhancing mutations from A.01 with stability-enhancing mutations from A.06 and A.09, created without the additional use of BayesDesign (Figure 3). This recombination approach yielded diverse functional profiles, with B.02 resulting in a significant increase in stability but lower activity than A.01 (Figure 2a). B.06 demonstrated improvements in stability and retained the activity of A.01, albeit with large stability error bars due to poor expression (see Figure S1). Additionally, we ran a second round of expert-guided deep learning that applied combinations of the masks used to generate A.06 and A.09 (mutating loop regions and areas dynamically correlated with the N-terminus, respectively) to variant A.01, which resulted in variants B.07–B.10 (Figure 2b, see Methods for more details). This approach yielded several exceptional variants as reported in Figure 2a. B.09 achieved the highest activity in our data set (172.1 ± 12.3% of wild-type) and maintained improved stability (16.1 ± 4.3% solubility). Variant B.07 closely matched this activity level (170.0 ± 16.4%) with slightly higher stability (18.0 ± 1.5% solubility).

**Figure 3:**
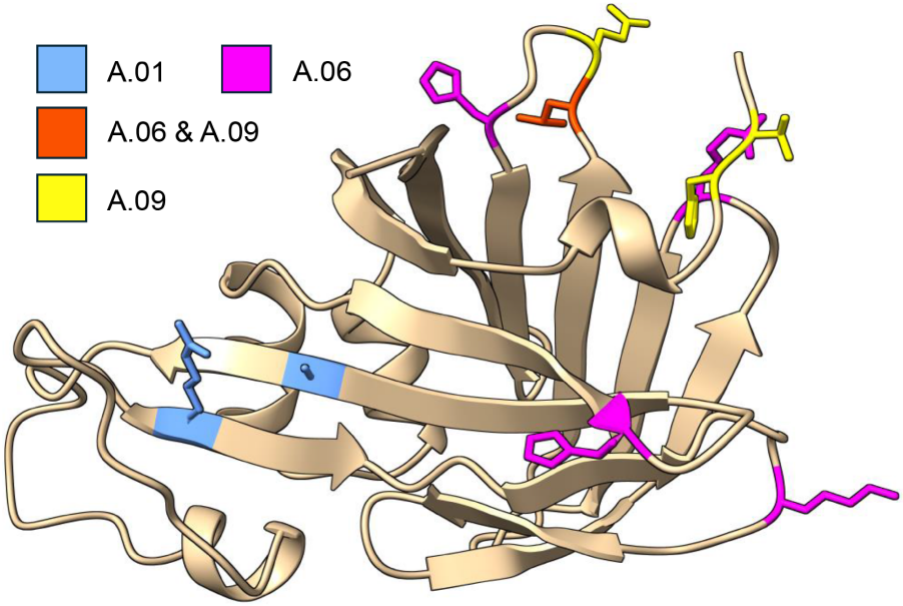
Structural and functional analysis of NanoLuc variants across libraries A and B. Mapping of beneficial mutations from library A that were recombined in library B to produce variants B.02–3, B.05–6, and B.09, visualized on the NanoLuc crystal structure (PDB ID: 7SNT). Key mutation sites are color-coded according to their parent sequences.

Overall, six of the nine library B variants exhibited substantial enhancements in stability compared with A.01, while simultaneously maintaining significantly increased enzymatic activity relative to wild-type NLuc. It should be noted that replacing the homology-based approach from the first three rounds with this rational design-based approach produced many variants with maintained activity. While previous rounds successfully produced soluble enzymes up to 95 °C, those enzymes lacked detectable activity. Results for libraries A and B indicate that targeting disordered regions (defined here as regions that are not alpha-helical or β-sheet) distant from the active site and enhancing dynamically coupled networks were particularly effective approaches for balancing stability and function. These findings highlight the complex nature of the stability-activity trade-off and how a modified approach combining both expert-guided design and deep learning can be used to mitigate it.

#### Computational Investigation of NLuc Variants

Figure 4a illustrates the mutational sequence space explored across libraries A and B, revealing distinct patterns in BayesDesign’s sampling strategy. Across most permissible positions, BayesDesign predominantly sampled only two amino acid variations, suggesting a focused convergence toward specific biochemical properties at these sites. This limited mutational diversity likely reflects the algorithm’s targeting of evolutionarily conserved positions, where only a narrow range of substitutions can occur without compromising structural integrity. Notably, variant B.09 stands as an exception to this pattern, exhibiting unique mutations at positions V2H and K125Q that were not observed in any other variant. These exclusive substitutions in B.09—replacing a hydrophobic valine with a positively charged histidine at position 2 and substituting a positively charged lysine with a polar glutamine at position 125—suggest the algorithm explored alternative electrostatic and hydrogen-bonding networks in this design iteration. The restricted mutational diversity at most positions, contrasted with these unique variations in B.09, highlights the delicate balance between conservation and innovation in our protein engineering approach, where BayesDesign effectively navigated the vast theoretical sequence space to identify viable candidates with enhanced functional properties.

**Figure 4:**
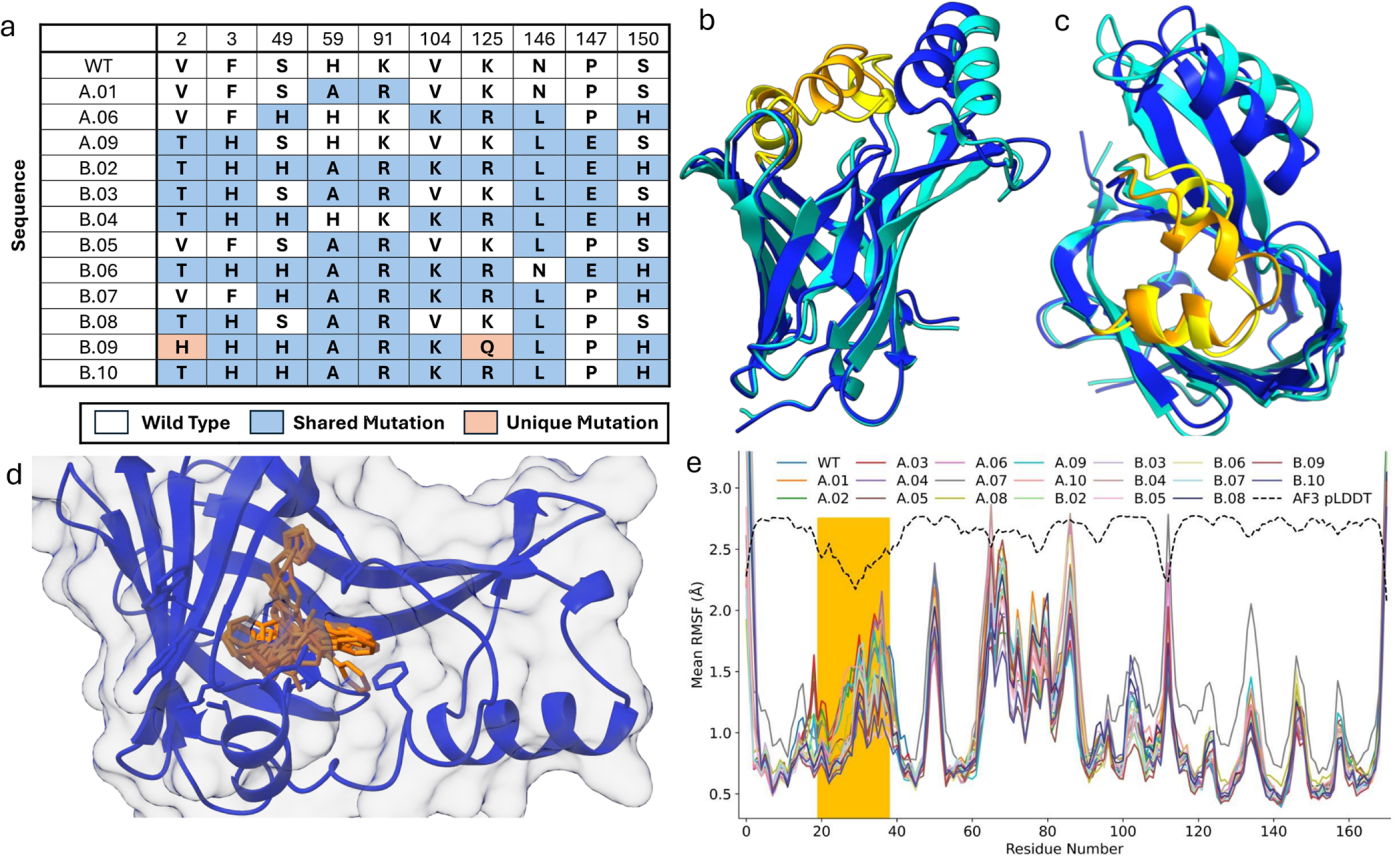
Computational analysis of mutants. a) Sequences of the most important mutants investigated. Top row indicates the residue position; letters stand for amino acid codes. b) The side and c) top views of two conformations assumed of all mutants when folded repeatedly with AF3 in absence of a substrate. Golden helix indicates area of high uncertainty in AF3 pLDDT scores. d) Predicted binding position of all FMZ substrates in one example backbone. e) C-alpha RMSF of all variants derived from MD simulations. The black dashed line shows a rescaled (divide by 100 add 1.5) pLDDT average score across all variants from AF3. The yellow highlight corresponds to the possible lid region highlighted in b) and c).

Structural ensemble analyses are presented in Figure 4b (side view) and Figure 4c (top view), revealing distinct conformational states sampled by AlphaFold (AF3).^32^ Remarkably, each mutant sequence consistently yielded two alternative conformational predictions with high confidence. As these structural models were generated in the absence of substrate, the dual conformations likely represent functionally relevant states that correspond to “open” and “closed” conformations of the enzyme. Of particular significance is the orange-coded loop region spanning residues 19–31, which contains transient helical motifs and exhibits the highest uncertainty in predicted Local Distance Difference Test (pLDDT) confidence scores across all AF3 predictions (see also Figure S2). This region of heightened conformational ambiguity appears to constitute a flexible lid domain that may regulate substrate access to NLuc’s beta-barrel active site. The conformational heterogeneity captured in these models aligns with established principles of enzyme dynamics, wherein flexible elements often modulate catalytic function through controlled transitions between discrete structural states. This computational evidence for conformational plasticity provides valuable insights into the structural basis of NLuc’s catalytic mechanism and offers a framework for understanding how mutations might influence the dynamic equilibrium between functional states.

Figure 4d provides a detailed examination of the binding site architecture across NLuc variants. We employed AF3 to model all mutant sequences in complex with the substrate FMZ, enabling assessment of potential changes in substrate-binding interactions. While conformational heterogeneity was observed comparable to that seen in apoprotein predictions, the location of the binding pocket remained remarkably consistent across all variants. Notably, FMZ possesses 10 energetically stable conformers,^33^ and AF3 predictions sampled a diverse spectrum of these rotameric states within the binding site. Given the recognized limitations of AF3 in accurately predicting fine-grained ligand-protein interactions, particularly for flexible small molecules, we emphasize the qualitative consistency in substrate docking behavior rather than specific interaction energies. The preservation of binding site topology across variants suggests that the mutations introduced by BayesDesign modulate protein stability and dynamics without fundamentally altering the substrate recognition mechanism. The subtle structural adaptations that likely underlie the observed enhancements in catalytic efficiency appear to be below the resolution threshold detectable by comparative structural modeling.^34^

Figure 4e illustrates the dynamic behavior of all 20 NLuc variants through 15 independent 10 ns molecular dynamics simulations each, with FMZ in the binding site (see also Figure S3). Throughout all simulations, FMZ remained stably bound in the active site without requiring additional constraints, validating the appropriate electrostatic environment for maintaining the noncovalent holo-conformation. Interestingly, the α-helical lid region exhibited reduced conformational fluctuations compared with what might be expected from the conformational heterogeneity observed in Figure 4b and c. This discrepancy likely stems from the presence of FMZ in the binding site, which appears to stabilize the “open” conformation and restricts the conformational sampling of the lid region during simulation time scales. We overlay in Figure 4e (dashed black line) the average pLDDT confidence scores from AF3 predictions across all mutants with bound FMZ, revealing a significant inverse correlation with the root-mean-square fluctuation (RMSF) values from MD simulations (Pearson correlation of −0.5, *p* < 0.001). This correlation suggests that regions with lower predicted structural confidence tend to exhibit greater dynamic flexibility. A notable exception occurs in the segment spanning residues 60–90, which displays elevated RMSF values despite relatively high pLDDT confidence scores. This region corresponds to a segment that was poorly resolved in the crystallographic structure (PDB ID: 7SNT), with electron density missing for 11 residues.

### Biophysical Characterization of Selected NLuc Variants

To better evaluate the most promising NLuc variants, we conducted further analyses of thermostability, pH dependence, spectral properties, and kinetics using the three most interesting sequences that resulted from the expert-guided deep learning approach described above—A.06, B.07, and B.09—and compared them with wild-type and A.01 sequences. For reference, time-course profiles for each variant are reported in Figure S8.

#### Thermostability Profiles

Thermal shift assays were conducted to determine the melting temperatures of the variants (Figure 5). The melting temperature is defined as the minimum of the inverse derivative of the fluorescence profile for a given sample. The measured melting temperatures demonstrate that variant A.06 exhibited a 6.8 °C increase in comparison to the wild-type sequence upon which it is engineered, and variants B.07 and B.09 exhibited increases of 7.2 and 5.1 °C in comparison to A.01, respectively. The significantly improved melting temperature observed with A.06 compared to the wild-type confirms the effectiveness of targeting disordered regions distant from the active site. In contrast, the reduced melting temperature observed with the high-activity variant A.01 is consistent with its compromised solubility at elevated temperatures reported in Figure 2. Importantly, the differences in melting temperatures reported in Figure 5 are consistent with the differences in percent solubility after incubation at 60 °C reported in Figure 2 for WT, A.01, A.06, B.07, and B.09. Further solubility experiments corroborated the results of the thermal shift assays (Table S3, Figure S8).

**Figure 5:**
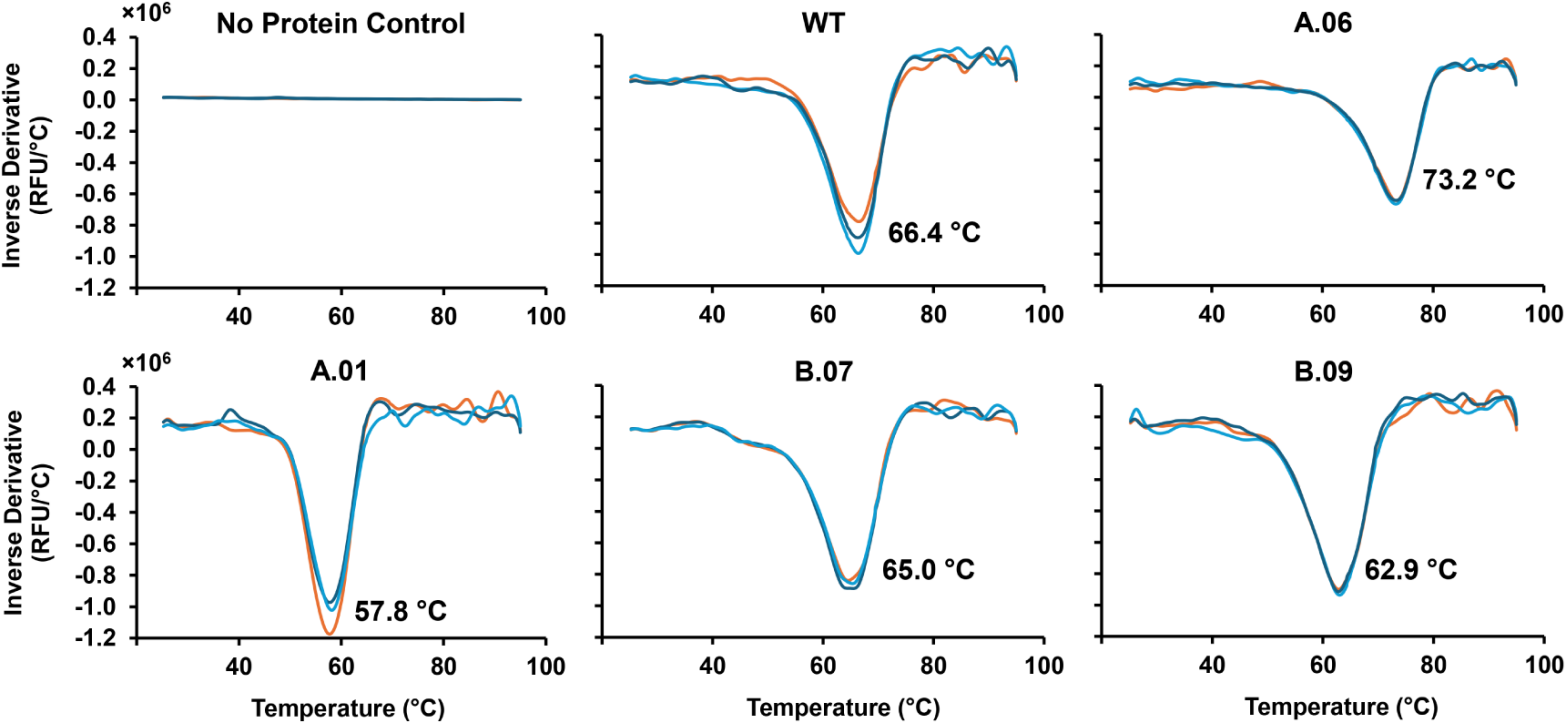
Thermal shift assay profiles of selected variants along with a no-protein negative control. The numerically labeled minimum of the inverse derivative of the fluorescent profiles is the melting point of the variant. Each line represents one replicate where each variant was sampled in triplicate.

#### Spectral Characteristics with Alternative Substrates

The spectral properties of the selected variants were examined with two distinct substrates—FMZ (the primary substrate for NLuc) and CTZ (an alternative substrate)—across the wavelength range of 400–600 nm (Figure S4, Table S4).

With CTZ as substrate, all variants maintained the characteristic, red-shifted emission profile compared to FMZ, with peak emission at approximately 480 nm compared to 460 nm (Table S5 and Figure S5). A.01 as well as the stability-enhanced variants of A.01 (B.07 and B.09) retained enhanced CTZ activity compared to the original NLuc.^18^

#### pH-Dependent Activity Profiles

The pH dependence of enzymatic activity across pH 4–9 was assessed to determine catalytic robustness under varying conditions (see Figure S4, Table S6). All variants displayed a pH-activity profile typical of luciferases, with activity increasing from acidic to neutral/basic conditions. Wild-type NLuc showed optimal activity at higher pH, with substantially reduced activity below pH 6. The high-activity variant A.01 maintained elevated activity across the pH range and retained significant activity at pH 5 (9.8 × 10^5^ RLU), representing a 5.3-fold activity increase over wild-type at this pH.

Variant A.06 exhibited a pH profile similar to the wild-type sequence. The hybrid variants B.07 and B.09 maintained both enhanced activity and pH tolerance inherited from A.01. B.09 achieved the highest overall activity, representing a 22% increase over wild-type at pH 9. Notably, all engineered variants showed minimal activity at pH 4, indicating that extreme acidic conditions remain challenging for NLuc function regardless of the thermostability-enhancing mutations.

#### Enhanced Catalytic Activity at Elevated Temperatures

An activity assay for the selected variants was run at 65 °C in the commercial Nano-Glo Luciferase Assay Buffer to determine the performance of the variants at elevated temperatures (Figure 6a).

**Figure 6:**
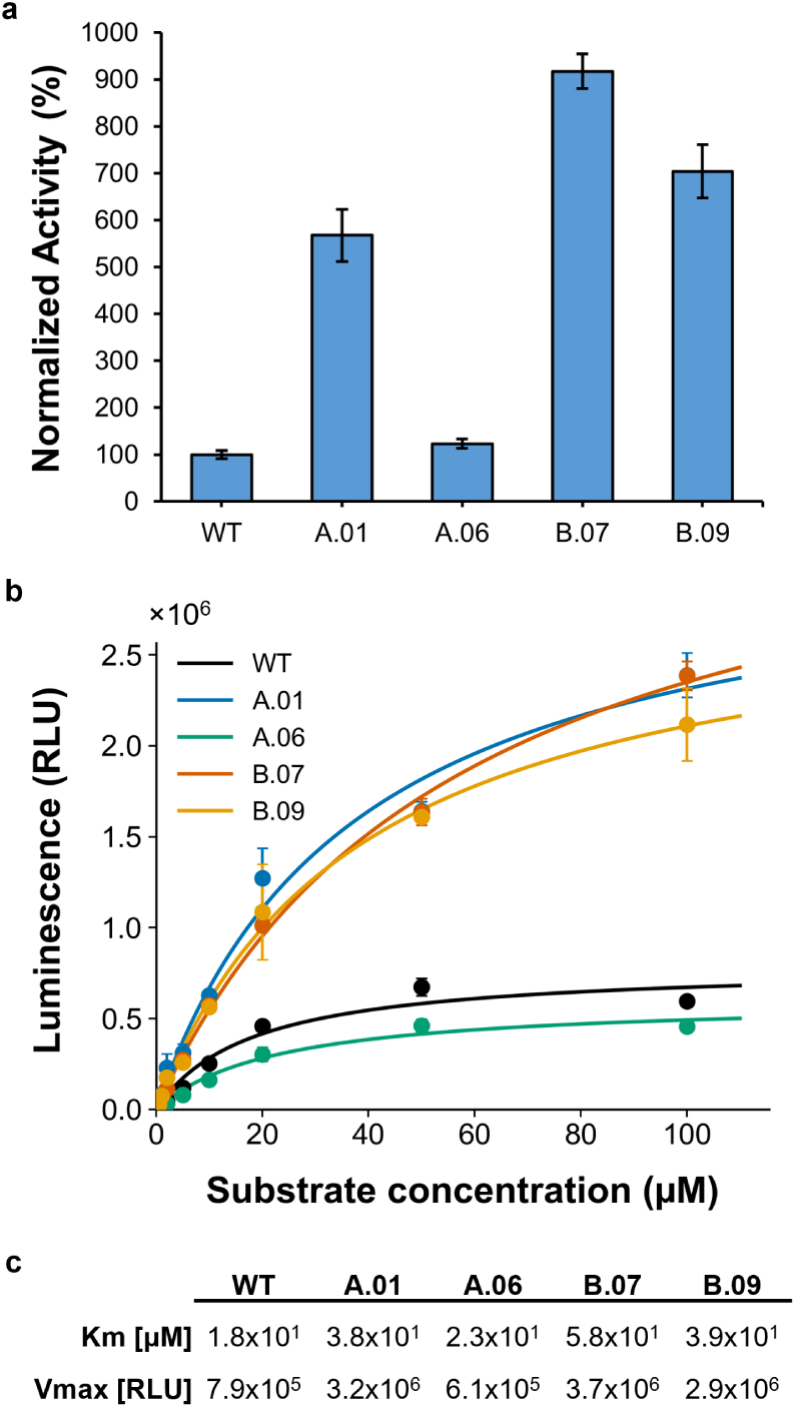
Activity and kinetics of wild-type NanoLuc and variants at elevated temperatures. a) Relative activity of wild-type and variants at 65 °C, taken at a single point and normalized to the activity of the wild-type at a final enzyme concentration of 0.50 nM. b) Initial reaction velocity versus substrate concentration for wild-type and variants toward FMZ at 55 °C at a final enzyme concentration of 5.0 nM. Values were fitted to the Michaelis–Menten equation. c) Table of kinetic parameters for wild-type and variants at 55 °C from b).

At 65 °C, variants B.07 and B.09 exhibited the highest activity at approximately 920% and 700% of wild-type activity, respectively, and 162% and 124% of A.01 activity, respectively (Figure 6a). All activity measurements in Figure 6a are statistically different (*p* < 0.05). This observed increase in activity compared to variant A.01 at 65 °C is attributed to the increased thermal stability of variants B.07 and B.09 compared to A.01 (see Figure 5) rather than improved catalytic activity. Variant A.06 exhibited 23% higher activity than wild-type, which is also attributed to increased thermal stability.

Kinetic parameters for the five selected variants were determined at 55 °C based on concentration-dependent luminescence in the commercial Nano-Glo Luciferase Assay Buffer (Figure 6b and c). At this temperature, A.01 and thermally stabilized variants based on A.01 (B.07 and B.09) exhibited significantly enhanced kinetic profiles compared to wild-type and A.06. Variants A.01, B.07, and B.09 displayed similar kinetic profiles while A.06 displayed a kinetic profile similar to wild-type. These results demonstrate that stabilizing mutations in the selected variants enhanced thermal stability without significantly reducing catalytic function under these conditions. This substantial performance enhancement at elevated temperatures highlights the potential application of these variants in higher-temperature sensing and industrial bioprocesses, where thermostable biocatalysts with enhanced catalytic efficiency could be beneficial.^19–21^

#### Effect of Freeze–Thaw Cycle on Enzyme Activity

Enzyme activity was measured at 55 °C after a single freeze–thaw cycle of the enzyme in phosphate-buffered saline (PBS) to assess resilience through comparative activity (Figure S7c). The results follow the trends of Figure 6a where the thermally stable variant A.06 is more resilient to a freeze–thaw cycle than the wild-type, and that variants B.07 and B.09 are likewise more resilient than A.01.

Collectively, these analyses demonstrate that our integrated expert-guided deep learning approach successfully generated NLuc variants with enhanced thermostability, while maintaining the catalytic properties at elevated temperatures, pH tolerance, and multisubstrate compatibility of the sequences upon which they were engineered. The performance of variants B.07 and B.09 highlights the effectiveness of our hybrid expert-guided deep learning engineering strategy in addressing the historically challenging trade-off between protein stability and activity.

## Conclusion

This study overcomes the previously reported stability-activity trade-off in NLuc luciferase engineering^22^ through an integrated expert-guided rational design and deep learning approach. By targeting disordered regions distant from the active site while preserving dynamically coupled networks, we developed variants with significantly improved thermostability (melting temperature increases up to 7.2 °C) while maintaining kinetic activity. These engineered luciferases maintain similar pH dependency as wild-type NanoLuc and improved compatibility with alternative substrates. Our hybrid methodology provides a potential framework for engineering the stability-activity trade-off for enzymes similar to NanoLuc with limited sequence homology, which could support the engineering of enhanced tools for bioluminescence imaging, biosensing, and protein interaction studies.

## Methods

### Generation of NLuc Variants

We followed the procedure outlined in Figure 7. NLuc variants were designed using BayesDesign, a deterministic deep learning algorithm that predicts the most probable sequence given structural and amino acid constraints. Structural constraints were defined by the Cα positions of wild-type NLuc, as specified in PDB ID: 7SNT, while amino acid constraints were based on the wild-type NLuc sequence, see Table S7 for all sequences. For each prediction, a subset of residues was selected for optimization, typically within flexible loop regions. BayesDesign was implemented via an established Google Colab notebook using sequence mask regions and input PDB structures as constraints.^22^ In rounds 4–5, sequences were generated using PDB ID: 7SNT as the target structure,^35^ while rounds 1–3 used PDB ID: 5IBO.^36^ Structures were obtained from the RCSB Protein Data Bank.^37^ Sequence generation followed a greedy decoding algorithm, proceeding from the N-terminus to the C-terminus. Positions designated as “fixed positions” remained unchanged during sequence optimization, ensuring the preservation of critical structural or functional elements.

**Figure 7:**
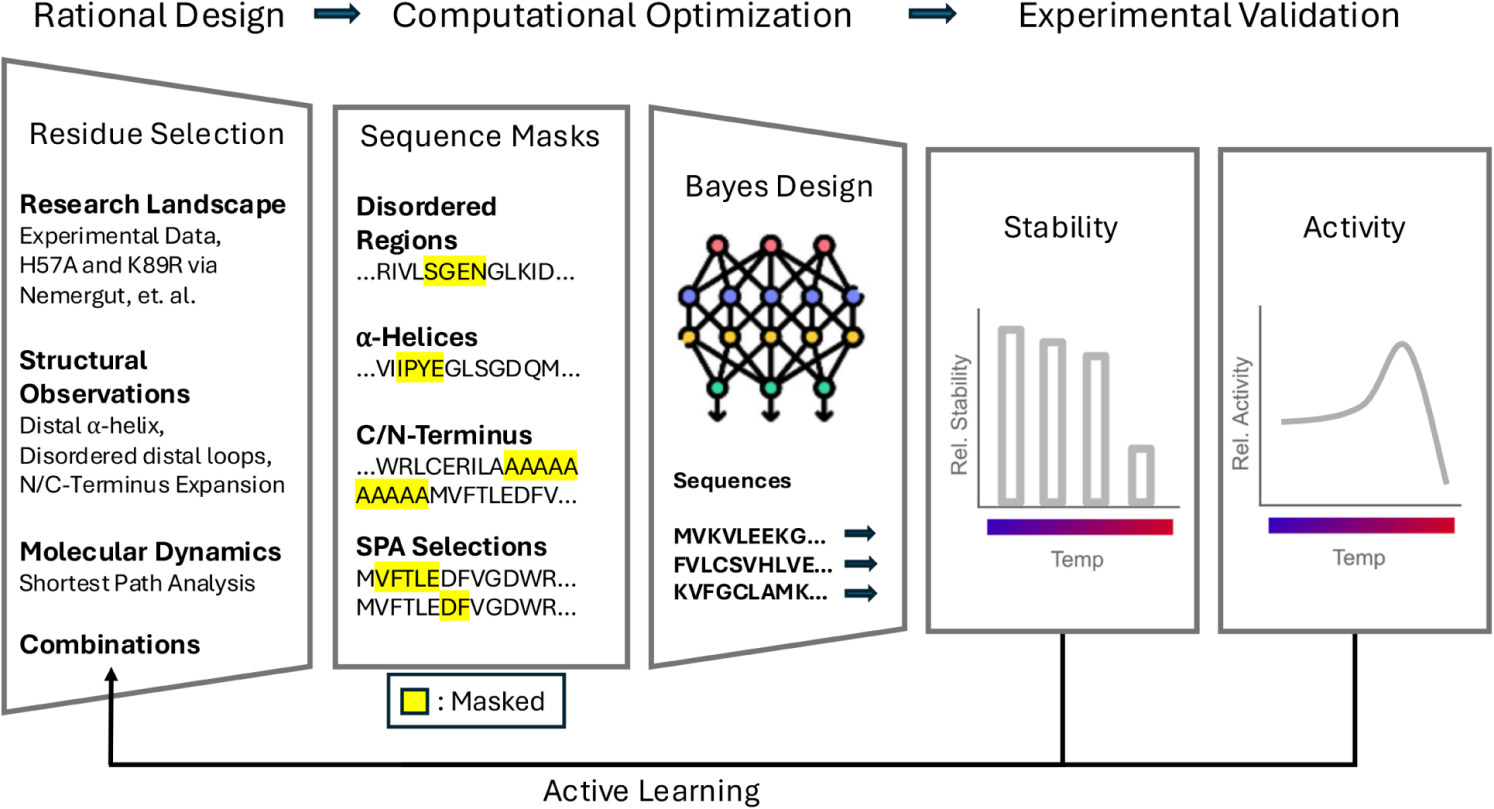
Integrated rational design and deep learning pipeline for enzyme engineering. Structure-guided analysis of literature and molecular simulations identifies key sequence regions for targeted optimization. The BayesDesign algorithm refines these sequences to discover variants with enhanced properties. Experimental validation provides feedback for subsequent rounds of expert-guided residue selection, creating an active learning cycle that efficiently navigates the sequence-function landscape.

### Generation of Variant Library A

Four sequences were selected based on combinations of mutations identified in previous studies: H59A and K91R from Nemergut et al.^18^ and V40T from Stern et al.^22^ The amino acid sequence numbering in this work is consistent with the original sequence reported by Hall et al.^16^ Sequence combinations are as follows: A.01 (H59A, K91R) and A.02 (V40T, H59A, K91R). Sequences A.03 and A.04 were constructed using Alanine substitutions in place of mutations identified in A.01 and A.02, respectively, making combinations: A.03 (H59A, K91A) and A.04 (V40A, H59A, K91A). Additionally, six sequences were designed by generating NLuc variants with BayesDesign, which entailed creating a mask (a list of residue positions that BayesDesign was allowed to mutate) and applying said mask to the wild-type sequence. These masks were created using a variety of rational design techniques, namely: helix regions (residues 62–65 and 71–84), loop regions (49–52, 101–106, 121–125, and 145–150), N-terminal additions (−4–0), C-terminal additions (172–176), dynamic correlations with the N-terminus (2–6, 146–147), and residues with minimal dynamic correlation (7, 8, 21, 27, 51, 63–64, 85–86, 99, 106, 120, 128, 145, 149). Dynamic correlations were identified via SPM analysis of MD simulations of the wild-type enzyme. Structures submitted to BayesDesign for terminal modifications were generated by appending alanine residues to the wild-type 7SNT structure in Chimera.^38^ For mutation details, see Table S8.

### Shortest Path Method Analysis

Analysis of molecular dynamics simulations from our previous work was conducted to inform the selection of mutation sites.^22^ The complex was prepared by docking FMZ with AutoDock Vina v1.2.3 to NLuc (PDB ID: 5IBO).^39^ Parameterization of FMZ used OpenFF’s SMIRNOFF force field^40^ while Amber ff14SB force field was used for the protein.^41^ Following solvation with explicit TIP3P water, charge neutralization, equilibration, minimization, and NPT equilibration, three independent 100 ns simulations were performed. The above processes were conducted using OpenMM’s molecular dynamics engine.^42^ All residues that came within 3 Å of the ligand in any MD simulation, as identified by ChimeraX 1.4,^36^ were considered active site residues. The resulting trajectories were analyzed using the web server developed by Casadevall et al.,^31^ which performs shortest path method (SPM) analysis. SPM analysis is an algorithm that uses MD trajectories to identify amino acids with correlated movements. Two SPM analyses were run, the first using default constraints (significance threshold 0.3, distance threshold 6). Visual inspection of the resulting contact map showed a correlation between the movements of residues 2–6 and 146–147. These positions defined the mask for variant A.09. The second analysis used maximally permissive constraints (significance threshold 0.1, distance threshold 10). The mask for variant A.10 was created by selecting residues that were not in the contact map and were not part of the active site.

### Generation of Variant Library B

Library B was derived from the best-performing sequences in library A, either combined directly (by maintaining mutations) or dynamically (by running BayesDesign with selected library A mutations as base sequences and applying the masking constraints of variants A.06 and A.09). Four direct combinations were generated as follows: B.02 (A.01, A.06, A.09), B.03 (A.01, A.09), B.04 (A.06, A.09), and B.05 (A.01, N146L). Sequence B.06 incorporated mutations from A.01, A.06, and A.09, except N146L, which was reverted to wild-type asparagine.

Four dynamic combinations were generated using a modified 7SNT structure as a backbone, using the ChimeraX rotamer tool^43–45^ to integrate mutations that define variants A.01 (for B.07–B.10) and A.06 (B.09) into the wild-type structure. These structures were submitted to BayesDesign with mutation positions defined as follows: B.07 (backbone: A.01, mask: A.06), B.08 (backbone: A.01, mask: A.09), B.09 (backbone: A.01, mask: A.06, A.09), and B.10 (backbone: A.01, A.06, mask: A.09). For mutation details, see Table S8.

### Structure Prediction

Protein structures were predicted using AF3.^32^ Model weights for self-hosted AF3 installation were made available by DeepMind upon our request—these were needed to fold variants with arbitrary substrates, like FMZ or CTZ. Each sequence in libraries A and B was input into a *json* file along with random seeds and processed by AF3. The random seeds allowed for analysis of the variations in conformation seen in Figures 4, S2 and S6. Each sequence produced five outputs ranked based on the highest average pLDDT confidence score. The output with the highest pLDDT score was used. Structures with the substrate FMZ were created by including the substrate in the same input file as the sequence. The output files from AF3 contained a pLDDT value for each atom within a structure. Here, the pLDDT values for each α-carbon were used.

ChimeraX 1.8 and 1.9 were used to visualize the AF3 outputs.^43–45^ pLDDT values were displayed throughout the structure with the AlphaFold Error Plot tool on ChimeraX. The Matchmaker tool was used in ChimeraX to align protein structures to compare sequences and substrate binding. To understand how the protein was interacting with the substrate, the Contact tool found in ChimeraX 1.9 was applied to the protein–substrate complex.

### Plotting and Visualization

Visualization and analysis were performed using Microsoft Excel, Matplotlib v3.7.1 ^46^ in Python v3.10, and UCSF ChimeraX 1.5.^43–45^ Experimental data in Figure 2a were visualized using Excel and flowcharts from Figure 2b in PowerPoint. Comparative analyses in Figures 3, 4e and 5 were generated using Matplotlib. Structural visualizations in Figures 3, 4b and d were rendered in ChimeraX using AF3-predicted protein models. Mutational effects in Figure 3 were modeled using the rotamer optimization tool in ChimeraX with Dunbrack 2010 rotamer libraries.^47^

### Protein Flexibility Analysis

Molecular dynamics (MD) simulations were performed using GROMACS 2024.3 with the CHARMM36m force field (July 2022 release).^48–56^ AF3-predicted structures of NLuc variants served as initial conformations. Substrate parametrization for FMZ was conducted using the CHARMM General Force Field (CGenFF v4.6) via the ParamChem web server,^57^ with partial charge assignments and dihedral parameters optimized according to established protocols. Ligand molecular structures were prepared and optimized using Open Babel v3.1.1 with the MMFF94s force field prior to parametrization.^58,59^

Each protein–ligand complex was centered in a dodecahedral simulation box with a minimum distance of 10 Å between the complex and box edges, then solvated with CHARMM-modified TIP3P water molecules.^32^ Systems were prepared with standard protonation states (N-terminus as NH_3_^+^, C-terminus as COO^–^) and neutralized by adding Na^+^ counterions to achieve electroneutrality. Energy minimization was performed using the steepest descent algorithm with a 0.01 nm step size and convergence criterion of maximum force below 1000 kJ mol^–1^ nm^–1^.

System equilibration consisted of sequential NVT and NPT ensemble simulations. The NVT step maintained 300 K for 100 ps using the velocity-rescaling thermostat (τ = 0.1 ps), followed by 100 ps NPT equilibration at 1 bar pressure controlled by the Berendsen barostat with stochastic cell rescaling (τ = 2.0 ps). Both steps used a 2 fs integration time step with the leapfrog algorithm. Long-range electrostatics were treated with the particle mesh Ewald (PME) method using a 1.2 nm real-space cutoff. van der Waals interactions were truncated at 1.2 nm with a smooth switching function applied from 1.0 nm. All bond lengths involving hydrogen atoms were constrained using the LINCS algorithm (fourth-order expansion with 1 iteration). The substrate was constrained with 1000 kJ mol^–1^ nm^–2^.

Production simulations were conducted for each of the 20 variants (wild-type and 19 engineered variants). For statistical robustness, we performed 7 independent 100 ns simulations per variant using different initial velocity distributions, yielding a cumulative 14 μs of simulation data. All production runs maintained NPT conditions (300 K, 1 bar) with identical parameters as the equilibration phase. Trajectories were saved every 10 ps for analysis.

Root mean square fluctuation (RMSF) values for Cα atoms were calculated using the GROMACS gmx rmsf module after least-squares fitting to the initial structure to remove translational and rotational motion.^51^ For each variant, RMSF profiles were averaged across all 15 independent simulations to ensure statistical significance and minimize bias from any single trajectory. These averaged RMSF values were then mapped onto the protein structures and correlated with experimental activity and stability measurements.

### Expression of NLuc Variants

Cell extract for protein synthesis was prepared from *E. coli* BL21-Star DE3 cells (Invitrogen, Carlsbad, CA, USA) in 2xYT media. Cell-free protein synthesis (CFPS) was performed as reported previously.^22,60^ Here, 1 L cultures were induced with isopropyl β-d-1-thiogalactopyranoside (IPTG) at an OD_600_ of 0.5–0.7 and harvested at an OD_600_ of 2–4. Cells were washed and then lysed with an Avestin Emulsiflex B-15 homogenizer (Avestin, Ottawa, Canada) at 21000 psi over 3 system passes. The lysate was then centrifuged for 30 min at 12000 RCF. The supernatant was collected and then used in CFPS reactions at 25% (v/v). PANOx-SP prepared as reported previously was added to CFPS reactions at 25% (v/v).^61^ The DNA template was added to CFPS reactions as unpurified polymerase chain reaction (PCR) product at 33% (v/v). Protein yield was determined by drying 2 μL of unpurified CFPS product on filter paper, washing the sample 3 times with TCA (trichloroacetic acid), and measuring the percentage of incorporated 14C-leucine (added at 5 μM concentration in the initial CFPS reaction preparation) using a liquid scintillation counter.^62^ Cell-free expression enables rapid production and single-step purification of variants which facilitated direct assessment of thermal stability and activity on the same day of synthesis, which can prevent the need for overnight storage, freeze–thawing, and additional homogenization and purification procedures required by *in vivo* expression. This enabled more accurate assessment of the original thermal stability and activity of NLuc and NLuc variants.^63–65^

### Activity and Stability Assays

NLuc mutants were expressed using CFPS for 3 h at 37 °C and 280 rpm. Solubility assessment was performed as previously reported.^22^ Following synthesis, each reaction was sampled for scintillation counting and then divided into 10–12 μL aliquots. Each aliquot was heat-treated within the range of 37–95 °C for 15 min in a PCR thermal cycler followed by centrifugation for 15 min at 16100 g and 4 °C. The supernatant was sampled in triplicate for scintillation counting to determine solubility. All activity assays were performed in a 96-well plate and measured in a Biotek (Winooski, VT, USA) SynergyMx plate reader, with a maximum reading temperature of 65 °C. Enzyme activity was determined by first diluting the protein 500x in 1x PBS buffer and then adding 2 μL of the protein dilution to 75 μL of assay reagents per well, which includes 34.7 μL 1x PBS, 39.5 μL Nano-Glo Luciferase Assay Buffer, and 0.79 μL Nano-Glo Luciferase Assay Substrate (Promega, Madison, Wisconsin, USA). Activity assays for rounds 1–3 and library A were performed at 30 °C, while activity for library B was run at 37 °C. For these activity assays, substrate was added, the well(s) were shaken for 30 s by the plate reader, and measurements were taken after waiting an additional 150 s (3 min after substrate addition). Default plate reader parameters were used.

After identifying the most promising mutants, final activity assays were conducted using the following formulations. Protein sequences from Figure 6a were formulated by diluting the protein in Nano-Glo Luciferase Assay Buffer to a concentration of 0.55 nM. 90 μL of protein was added to a 96-well plate then preheated to 65 °C for 15 min. A 1 mM stock solution of FMZ (DC Chemicals, Shanghai, China) was prepared by diluting solid FMZ into ice-cold Nano-Glo Luciferase Assay Buffer. Then, 10 μL of the stock solution was added to the wells at a final concentration of 100 μM. The activity assay was run at 65 °C, following the measurement protocol detailed above. CTZ sensitivity was determined by diluting protein 500x in 1x Dulbecco’s PBS. Two μL of diluted protein was added to 75 μL of assay reagents, which include 34.7 μL 1x Dulbecco’s PBS, 39.5 μL Gaussia Glow Assay Buffer, and 0.79 μL 100x Coelenterazine (Pierce Gaussia Luciferase Glow Assay Kit, Thermo Scientific, Waltham, MA, USA). CTZ luminescence was measured at 37 °C, following the measurement protocol detailed above.

Thermal shift assays were performed as reported previously.^66^ Proteins were first purified using the Strep-Tactin XT 4Flow high capacity Spin Column Kit (IBA Life Sciences, Gottingen, Germany) as specified by the manufacturer. The columns were washed 2 times with Buffer W, before eluting to the specified high-concentration option. Liquid scintillation counting was used to determine purified protein yields. Melting temperature was determined using the Protein Thermal Shift Dye Kit (Thermo Fisher Scientific, Carlsbad, CA), which uses a hydrophobic dye that fluoresces when bound to hydrophobic regions of a protein upon denaturation. The thermal shift reactions contained the following composition: 5 μL Protein Thermal Shift Buffer, 2.5 μL Diluted Protein Thermal Shift Dye (8×) diluted with water, and 12.5 μL Strep-TactinXT Elution Buffer (Buffer BXT) containing 500 ng of purified protein sample. Reactions were performed in triplicate in a FrameStar 96-Well Semi-Skirted PCR Fast Plate (Midsci, St. Louis, MO) and covered with a MicroAmp Optical Adhesive Film (Applied Biosystems, Thermo Fisher Scientific). The assay was run in a StepOnePlus Real-Time PCR System (Applied Biosystems) at standard ramp speed covering a range of 25–95 °C.

### Kinetic Parameter Assay

Kinetic parameters were determined by measuring the luminescence of selected NLuc variants under FMZ concentrations ranging from 0.1 to 100 μM. FMZ solutions were prepared by diluting the 1 mM stock solution described previously into Nano-Glo Luciferase Assay Buffer. Measurements were taken at 55 °C in a Biotek SynergyMx plate reader where 10 μL of substrate solution were added to 90 μL of preheated enzyme (at a final enzyme concentration of 5.0 nM in a 96-well plate). Initial reaction velocity was calculated as the raw luminescence value measured 15 s after substrate addition to capture the linear stage of the reaction. This 15-s interval included a 10-s shaking period by the plate reader. Default plate reader parameters were used with a 1-s integration time; therefore, relative luminescent units (RLU) are proportional to photon generation per second. Initial reaction velocities were fit to the Michaelis–Menten equation using GraphPad Prism 10.5.0 (GraphPad Software, USA). Reactions were performed in triplicate. It should be noted that the 100 μM data points in Figure 6b were obtained from the initial time points (*t* = 0) measured during the time-course evaluation of selected variants (see Figure S8). All other data points used in this assay were measured independently.

### pH Assay

The pH assay buffers were prepared according to previously reported protocols,^16^ where buffers contained 100 mM citrate, HEPES, or tricine, 1 mM DTT, 10 mM MgSO4, 0.5% Tergitol, and 0.05% (v/v) Antifoam 204, titrated to their respective pH values. For the pH assay, proteins were diluted 500x into their respective pH buffers, then added to 75 μL of assay reagents per well, which includes 74.21 μL assay buffer and 0.79 μL of Nano-Glo Luciferase Assay Substrate. Activity was measured at 37 °C 3 min after substrate addition, following the activity assay measurement protocol.

### Emission Range Verification

Emission spectra were measured from 400 to 600 nm wavelengths at 10 nm intervals with the Biotek SynergyMx plate reader at 30 °C.

## Supplementary Material



## Notes

Table S1: Numerical results of solubility and furimazine activity measures for 5 rounds of NLuc design. Figure S1: Library B protein yields. Figure S2: Structural variability and confidence in AlphaFold 3 predictions for NLuc variants. Figure S3: Analysis of molecular dynamics simulations. Figure S4: pH profile and emissions spectra of selected NLuc variants. Figure S5: Average activity toward CTZ of selected NLuc variants. Table S2: Normalized stability across temperatures. Table S3: T50 for selected NLuc variants. Table S4: Emission intensity across wavelengths for CTZ. Table S5: Emission intensity across wavelengths for FMZ. Table S6: Activity across pH. Figure S6: Alphafold3 per-residue confidence scores. Table S7: Sequences for Library A and Library B. Table S8: Mutational details. Figure S7: Solubility and post-freeze–thaw activity of promising NLuc variants. Figure S8: Time–course profiles. Supplementary Methods and Results (PDF)
