# Supplementary material for: Advancing NanoLuc Luciferase Stability beyond Directed Evolution and Rational Design through Expert-Guided Deep Learning
Source: ACS Catal. 2026 Jan 27;16(3):2849–60. doi: 10.1021/acscatal.5c08789 (PMC12887937; doi:10.1021/acscatal.5c08789)
Supplement: Supplementary file 1 [file cs5c08789_si_001.pdf]

Supporting Information

Spencer Gardiner[1,#], Joseph Talley[2,#], Tyler Green[2], Christopher Haynie[1], Corbyn Kubalek[1], Matthew Argyle[1], William Heaps[1], Joshua Ebbert[1], Deon Allen[1], Dallin Chipman[2], Bradley C Bundy[2,], Dennis Della Corte[1,*]*

[1]Department of Physics and Astronomy, Brigham Young University, 84602 Provo

[2]Department of Chemical Engineering, Brigham Young University, 84602 Provo

**corresponding author computations: Dennis.DellaCorte@byu.edu*

**corresponding author experiments: bundy@byu.edu*

[#]these authors contributed equally

Table of Contents

Table S1. Numerical results of solubility and furimazine activity measures for 5 rounds of NLuc design. **.......... 2**

Figure S1. Library B protein yields. **.......... 4**

Figure S2. Structural variability and confidence in AlphaFold 3 predictions for NLuc variants **.......... 5**

Figure S3. Analysis of molecular dynamics simulations. **.......... 7**

Figure S4. pH profile and emissions spectra of selected NLuc variants. **.......... 7**

Figure S5. Average activity towards CTZ of selected NLuc variants. **.......... 8**

Table S2. Normalized stability across temperatures. **.......... 9**

Table S3. T_{50} for selected NLuc variants. **.......... 9**

Table S4. Emission intensity across wavelengths for CTZ. **.......... 10**

Table S5. Emission intensity across wavelengths for FMZ. **.......... 11**

Table S6. Activity across pH. **.......... 11**

Figure S6. Alphafold3 per-residue confidence scores. **.......... 12**

Table S7. Sequences for Library A and Library B. **.......... 12**

Table S8. Mutational details. **.......... 16**

Figure S7. Solubility and post-freeze-thaw activity of promising NLuc variants **.......... 18**

Figure S8. Time-course profiles. **.......... 19**

Supplementary Methods & Results **.......... 19**

- **Freeze-thaw Method 19**
- **Freeze-thaw Results 19**
- **Time-Course Methods 19**
- **Time-Course Results 20**

References **.......... 20**

Table S1. Numerical results of solubility and furimazine activity measures for 5 rounds of NLuc design.

Variant Name	Sequence Homology [%]	Solubility Mean [%]	Solubility Std [%]	Mean Activity [%]	Activity Std [%]
Wild-type	100	9.72	1.24	100.00	13.72
1.01	32	0.00	NA	0.00	NA
1.02	36	0.00	NA	0.00	NA
1.03	34	0.00	NA	0.00	NA
1.04	34	0.00	NA	0.00	NA
1.05	32	11.78	NA	0.00	NA
1.06	30	0.00	NA	0.00	NA
1.07	29	0.00	NA	0.00	NA
1.08	31	0.00	NA	0.00	NA
1.09	33	1.72	NA	0.00	NA
1.10	34	0.00	NA	0.21	NA
2.01	48	79.45	3.33	0.20	0.05
2.02	60	84.60	5.95	0.03	0.19
2.03	72	94.58	4.99	0.04	0.35
2.04	62	91.89	2.34	0.01	0.15
2.05	60	94.67	5.75	0.05	0.12
2.06	69	80.20	6.46	0.02	0.05
2.07	67	94.74	7.17	0.01	0.03
2.08	73	93.28	8.37	0.05	0.39
2.09	96	12.43	0.74	41.29	5.33
3.01	99	54.45	1.17	0.02	0.00
3.02	99	77.31	1.09	0.00	0.00
3.02	99	64.91	3.55	0.01	0.00
A.05	94	5.86	0.99	82.09	7.82
A.06	97	71.83	9.62	102.03	10.88
A.07	100	12.62	2.38	105.57	6.03
A.08	100	3.73	0.52	98.82	15.81
A.09	98	40.14	5.63	95.38	36.87
A.10	98	3.07	1.71	0.02	0.00
B.04	95	64.01	11.27	82.42	3.15
A.01	99	1.49	0.52	169.37	5.83

A.02	98	1.48	0.30	54.79	2.23
A.03	99	1.30	0.63	131.55	12.27
A.04	98	2.88	1.22	17.31	2.17
B.02	94	27.60	2.25	127.92	9.92
B.03	97	3.10	2.06	92.95	1.78
B.05	98	1.20	0.28	150.49	7.99
B.06	95	26.74	18.71	162.10	14.37
B.07	96	18.03	1.46	170.04	16.40
B.08	97	11.00	14.44	139.46	6.57
B.09	95	16.09	4.27	172.05	12.25
B.10	95	23.07	2.67	147.18	14.37

**Variants starting at A.01 used the sequence of A.01 as starting point. All other variants (listed above A.01) were based on wild-type sequence.*

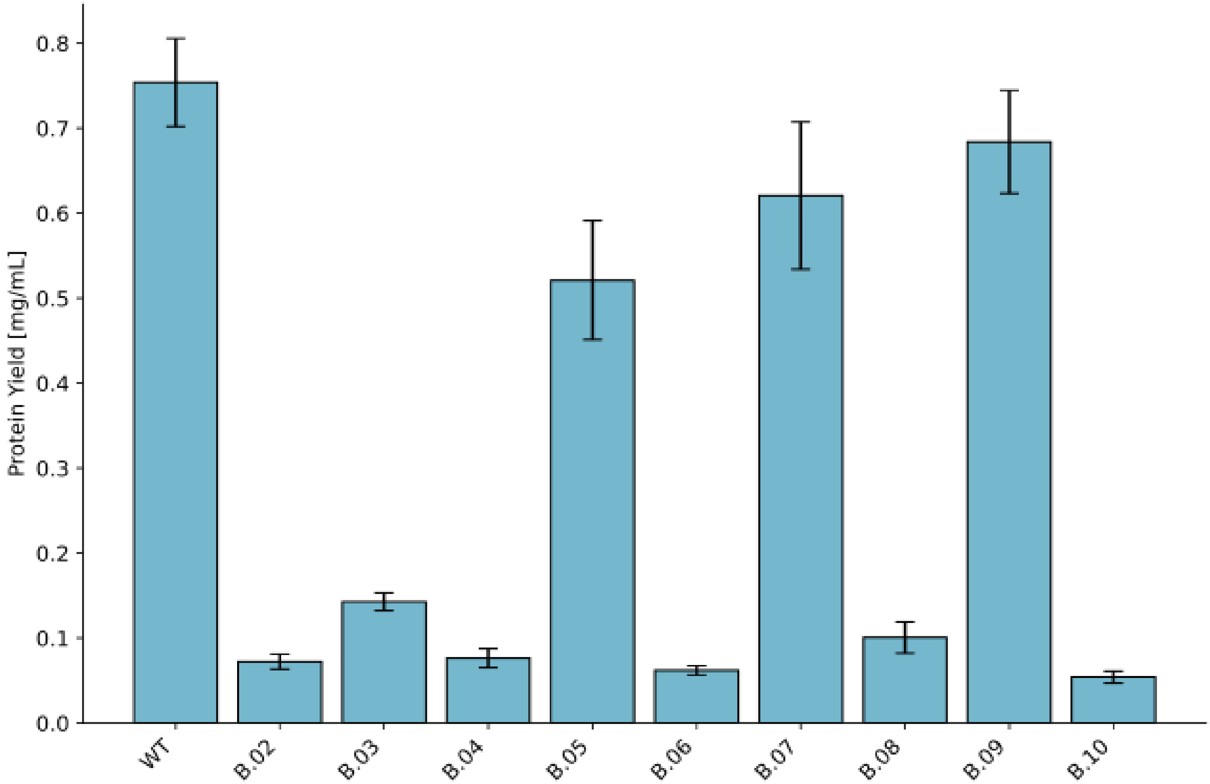

Figure S1. Library B protein yields. *Variants with the mutation V2T had significantly lower yields than variants without that mutation.*

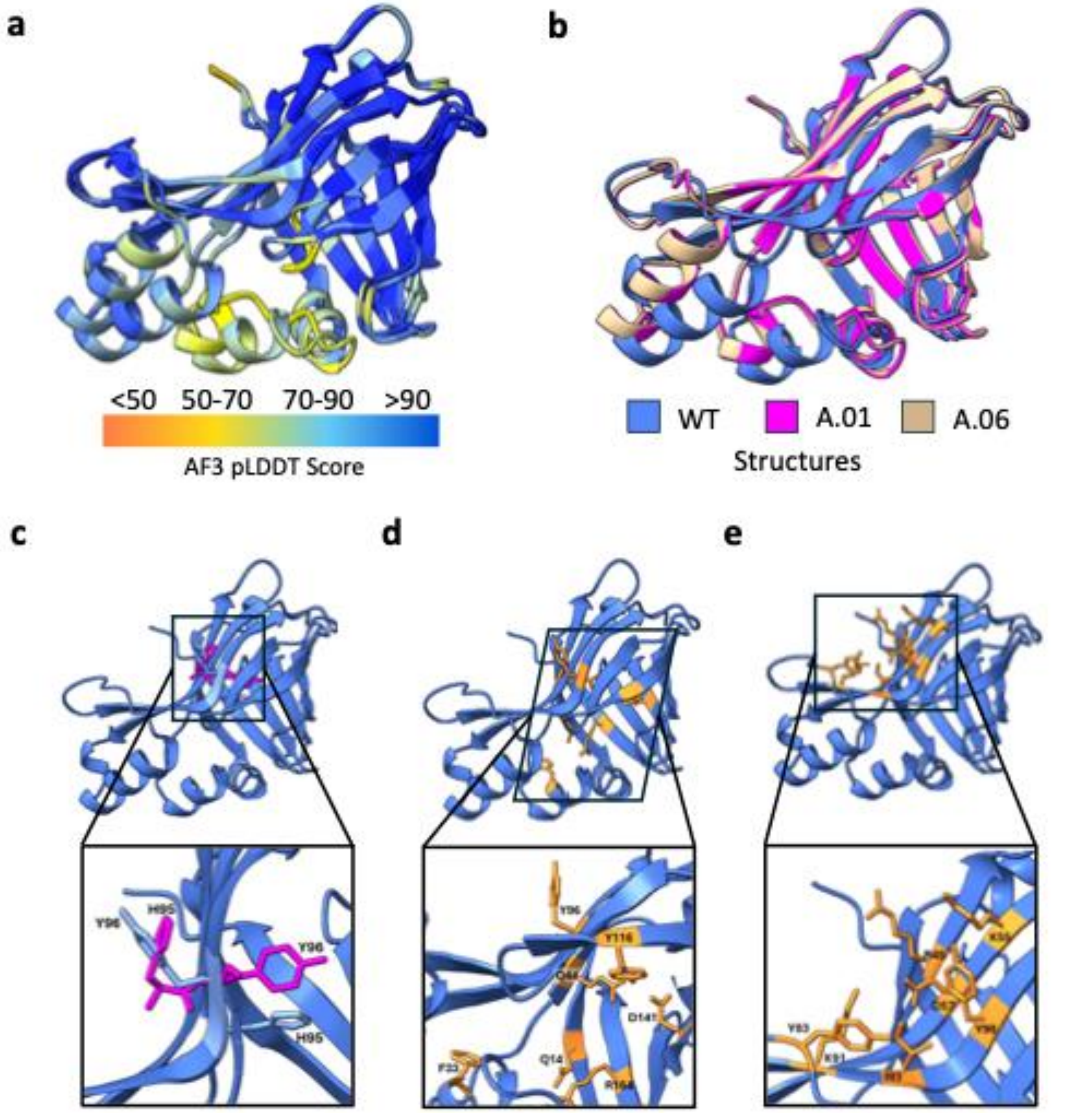

Figure S2. Structural variability and confidence in AlphaFold 3 predictions for NLuc variants. *a) Overlay of predicted structural confidence, shown as per-residue pLDDT scores. b) Overlaid representative conformations of selected mutants generated using different AlphaFold 3 random seeds. c) Conformations and orientations of side chains of H95 and Y96 residues, showing both open and closed conformations sampled by AF3 [1]. No correlation was observed between mutant stability or activity and the conformation of predicted AF3 structures. d), e) Catalytic and allosteric sites of selected mutants.*

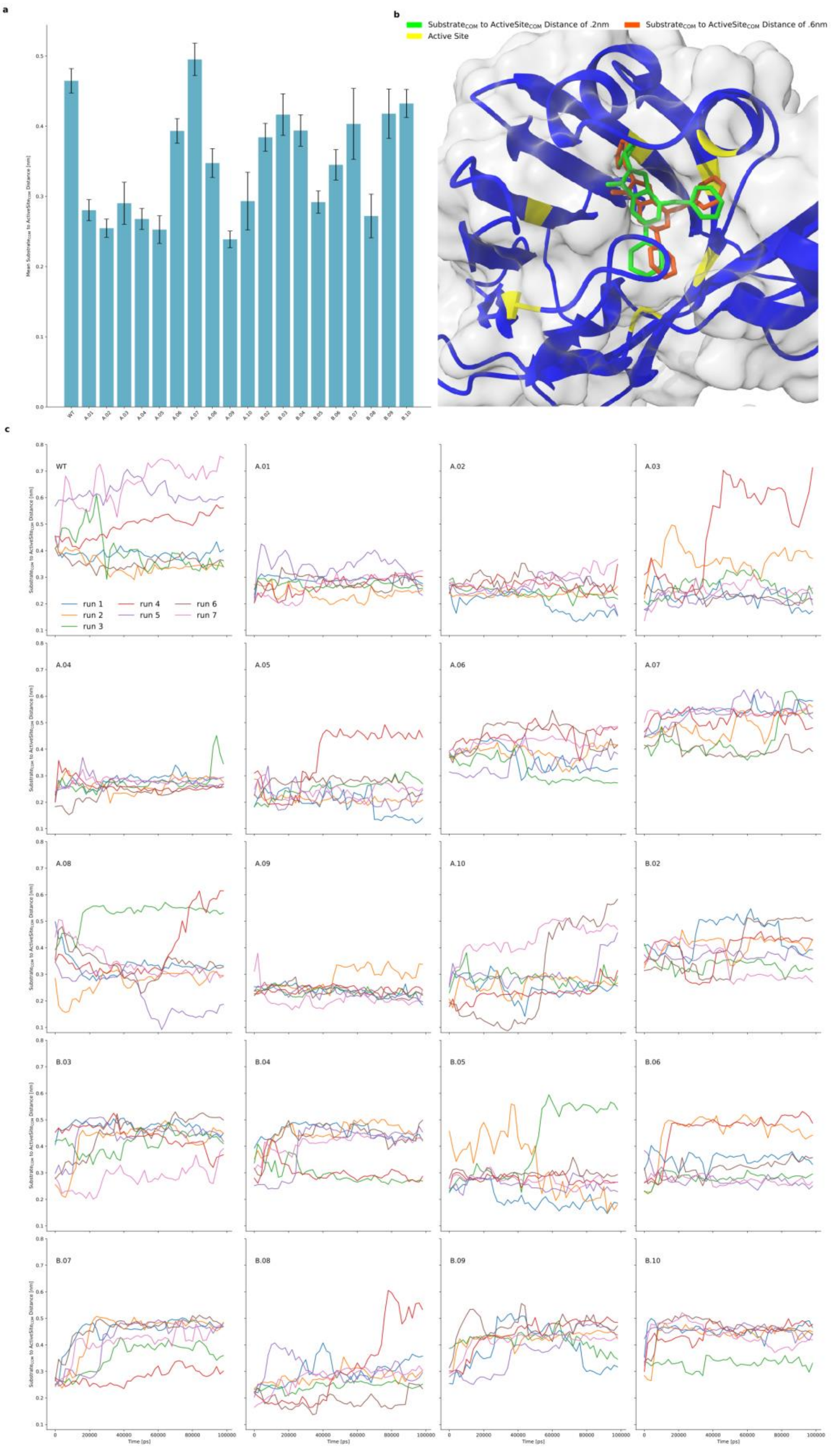
a
Mean Substrate$_{COM}$ to ActiveSite$_{COM}$ Distance [nm]
WT
A.01
A.02
A.03
A.04
A.05
A.06
A.07
A.08
A.09
A.10
B.02
B.03
B.04
B.05
B.06
B.07
B.08
B.09
B.10
b
Substrate$_{COM}$ to ActiveSite$_{COM}$ Distance of .2nm
Substrate$_{COM}$ to ActiveSite$_{COM}$ Distance of .6nm
Active Site
c
Substrate$_{COM}$ to ActiveSite$_{COM}$ Distance [nm]
run 1
run 2
run 3
run 4
run 5
run 6
run 7
Time [ps]

Figure S3. Analysis of molecular dynamics simulations. *a) Mean distance between the center of mass of the substrate and active site with the standard deviation as error bars. b) Modeling of two frames in the MD simulation. The green ligand is a frame where the distance between the center of mass of the substrate and active site is 0.2 nm and the orange ligand is a frame where that distance is 0.6 nm. c) Tracks the distance between the center of masses across the MD simulation.*

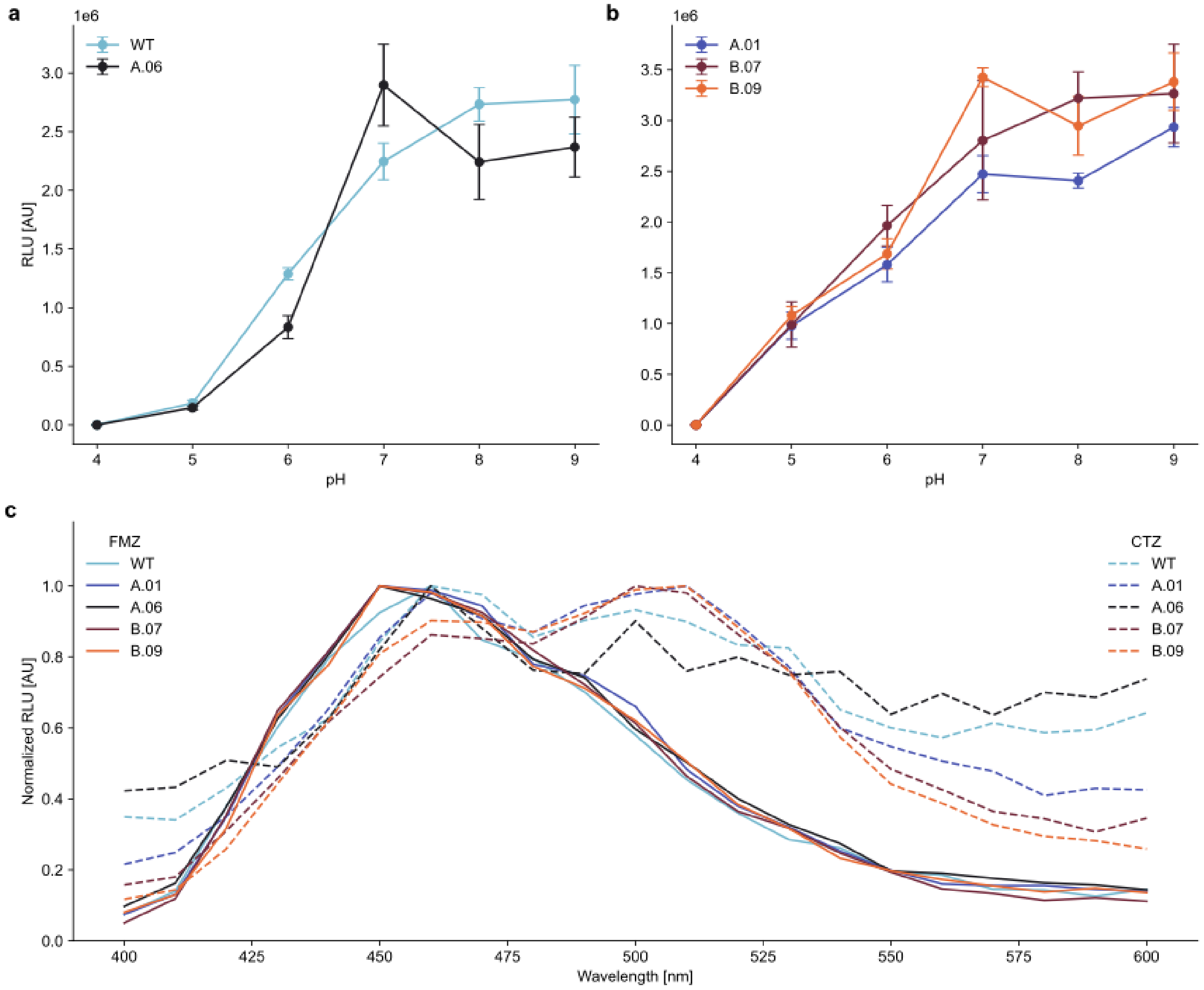

Figure S4. pH profile and emissions spectra of selected NLuc variants. *Experimental characterization of promising NLuc variants. a) pH-dependent bioluminescence profiles of variant A.06 compared to wild-type (WT). b) pH-dependent light output of A.01-derived variants. c) Normalized emission spectra for NLuc variants with substrates furimazine (FMZ) and coelenterazine (CTZ).*

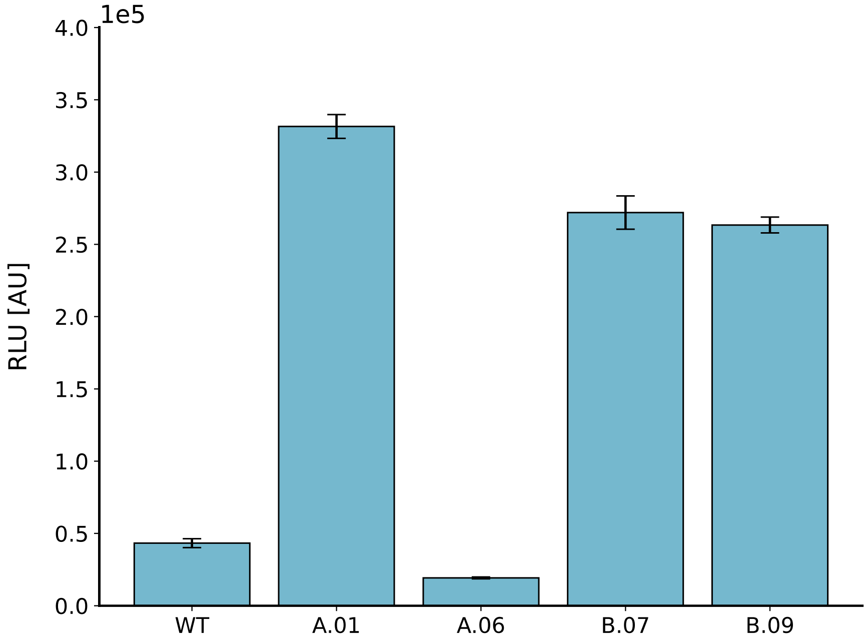

Figure S5. Average activity towards CTZ of selected NLuc variants. *Measured at 37 °C.*

Table S2. Normalized stability across temperatures. *(Mean ± Std)*

Variant	37 °C	40 °C	45 °C	50 °C	55 °C	60 °C	63 °C
WT	100.00 ± 9.48 %	108.85 ± 6.64 %	97.05 ± 6.54 %	85.03%± 4.85 %	49.69 ± 2.71 %	7.38 ± 0.41 %	1.25 ± 0.58 %
A.01	100.00 ± 23.00 %	119.00 ± 20.85 %	89.99 ± 12.22 %	62.13 ± 4.38 %	6.83 ± 0.64 %	0.88 ± 0.07 %	1.04 ± 0.84 %
A.06	100.00 ± 11.41 %	105.19 ± 12.02 %	98.91 ± 10.92 %	88.24 ± 9.83 %	97.58 ± 13.16 %	83.44 ± 11.19 %	59.12 ± 7.22 %
B.07	100.00 ± 10.61 %	84.45 ± 8.76 %	74.95 ± 9.45 %	69.98 ± 8.62 %	51.84 ± 5.17 %	17.01 ± 1.84 %	4.43 ± 1.92 %
B.09	100.00 ± 4.02 %	96.04 ± 5.63 %	92.89± 7.79 %	96.75 ± 7.30 %	61.70 ± 3.56 %	20.93 ± 3.65 %	2.81 ± 0.35 %

Table S3. T_{50} for selected NLuc variants. *Temperature where variants reach 50% solubility.*

Variant	T_{50} (°C)
WT	54.97
A.01	51.14
A.06	63.88
B.07	55.30
B.09	56.33

Table S4. Emission intensity across wavelengths for CTZ. (RLU)

Wavelength [nm]	WT	A.01	A.06	B.07	B.09
400	3749	3955	3628	4071	4175
410	3654	4552	3716	4643	5127
420	4621	6427	4371	8001	9251
430	5840	8971	4207	11787	15765
440	6718	11967	5383	15915	22124
450	9045	15642	7048	19176	28848
460	10723	17927	8583	22230	32141
470	10458	16610	7561	21959	31987
480	9178	15904	6545	21584	31014
490	9680	17283	6461	23479	32891
500	9999	17866	7736	25778	35256
510	9642	18297	6522	25269	35630
520	8942	16397	6862	22280	31513
530	8849	14141	6431	19677	27058
540	6989	10980	6515	15525	20492
550	6440	10014	5482	12474	15745
560	6140	9269	5978	10982	13810
570	6584	8740	5466	9398	11654
580	6287	7492	6010	8873	10501
590	6380	7867	5890	7920	10053
600	6892	7782	6335	8909	9234

Table S5. Emission intensity across wavelengths for FMZ. *(RLU)*

Wavelength [nm]	WT	A.01	A.06	B.07	B.09
400	1762	1984	2019	1937	2011
410	3313	3488	3393	4549	3254
420	8966	9322	7864	13590	7946
430	14206	16913	13003	24821	15916
440	18801	21708	16781	31276	19452
450	21841	26609	20807	38282	25006
460	23612	26285	20063	37518	24566
470	20019	25118	19074	35422	22903
480	18766	20739	16511	31355	19334
490	16570	19891	15438	27656	17816
500	13694	17573	12412	23486	15515
510	10735	12862	10472	17774	12650
520	8519	10109	8351	13974	9618
530	6742	8533	6825	12172	7932
540	6181	6733	5713	9483	5833
550	4605	5286	4092	7412	4940
560	4380	4277	3963	5601	4325
570	3428	4146	3667	5115	3876
580	3392	4156	3406	4367	3449
590	2970	3851	3286	4652	3715
600	3429	3726	2999	4265	3396

Table S6. Activity across pH. *(RLU ± SE)*

Variant	pH 4	pH 5	pH 6	pH 7	pH 8	pH 9
WT	1277 ± 242	185438 ± 25410	1288065 ± 51576	2245930 ± 155926	2732942 ± 142634	2772872 ± 290799
A.01	3731 ± 385	978691 ± 133330	1580074 ± 168181	2471771 ± 181974	2407249 ± 73990	2934566 ± 192842
A.06	0 ± 0	146170 ± 18231	833886 ± 98090	2896638 ± 348346	2241316 ± 319625	2368008 ± 254914
B.07	2669 ± 346	990072 ± 223761	1965307 ± 199285	2803502 ± 587333	3218153 ± 261742	3264504 ± 485724
B.09	3344 ± 426	1080999 ± 89427	1686998 ± 147955	3425378 ± 92665	2946734 ± 288716	3380240 ± 282937

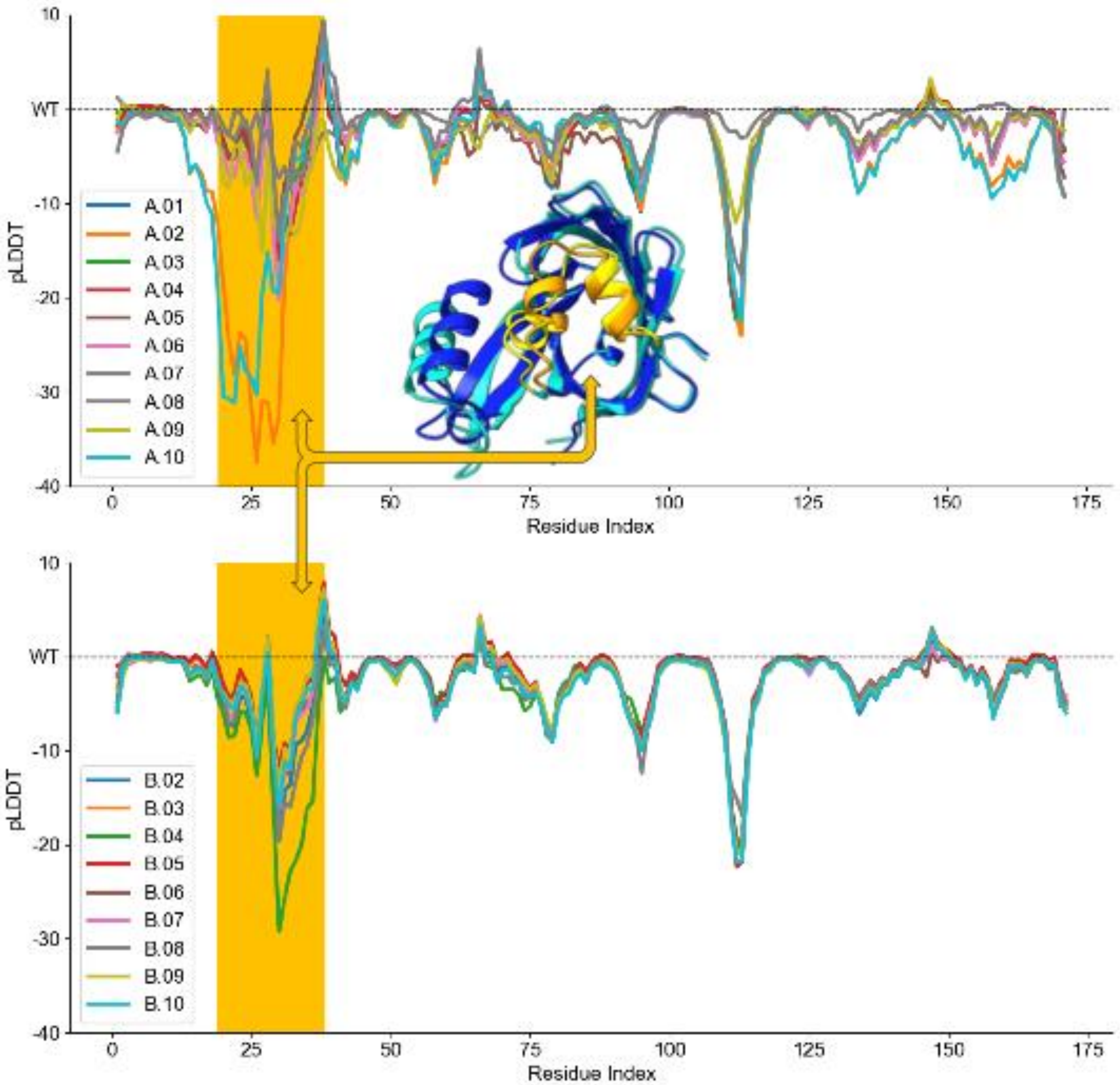

Figure S6. Alphafold3 per-residue confidence scores. *AlphaFold 3 pLDDT confidence score differences for library A (top) and library B (bottom). Per-residue pLDDT scores for each mutant were normalized by subtracting the corresponding wild-type (WT) scores to highlight regions of structural uncertainty. The putative lid domain, shown in orange in the inset structure, corresponds to the region with the greatest fluctuation across variants.*

Table S7. Sequences for Library A and Library B.

Sequence ID	Sequence

A.01	MVFTLEDFVGDWRQTAGYNLDQVLEQGGVSSLFQNLGVSVTPIQRIVL SGENGLKIDIAVIIPYEGLSGDQMGQIEKIFKVVYPVDDHHFRVILHYGT LVIDGVTPNMIDYFGRPYEGIAVFDGKKITVTGTLWNGNKIIDERLINPD GSLLFRVTINGVTGWRLCERILA
A.02	MVFTLEDFVGDWRQTAGYNLDQVLEQGGVSSLFQNLGVSTTPIQRIVL SGENGLKIDIAVIIPYEGLSGDQMGQIEKIFKVVYPVDDHHFRVILHYGT LVIDGVTPNMIDYFGRPYEGIAVFDGKKITVTGTLWNGNKIIDERLINPD GSLLFRVTINGVTGWRLCERILA
A.03	MVFTLEDFVGDWRQTAGYNLDQVLEQGGVSSLFQNLGVSVTPIQRIVL SGENGLKIDIAVIIPYEGLSGDQMGQIEKIFKVVYPVDDHHFAVILHYGT LVIDGVTPNMIDYFGRPYEGIAVFDGKKITVTGTLWNGNKIIDERLINPD GSLLFRVTINGVTGWRLCERILA
A.04	MVFTLEDFVGDWRQTAGYNLDQVLEQGGVSSLFQNLGVSATPIQRIVL SGENGLKIDIAVIIPYEGLSGDQMGQIEKIFKVVYPVDDHHFAVILHYGT LVIDGVTPNMIDYFGRPYEGIAVFDGKKITVTGTLWNGNKIIDERLINPD GSLLFRVTINGVTGWRLCERILA
A.05	MVFTLEDFVGDWRQTAGYNLDQVLEQGGVSSLFQNLGVSVTPIQRIVL SGENGLKIDIHVILSKDGLSGDQDQEIKKVFKHIYPVDDHHFKVILHYGT LVIDGVTPNMIDYFGRPYEGIAVFDGKKITVTGTLWNGNKIIDERLINPD GSLLFRVTINGVTGWRLCERILA
A.06	MVFTLEDFVGDWRQTAGYNLDQVLEQGGVSSLFQNLGVSVTPIQRIVL HGENGLKIDIHVIIPYEGLSGDQMGQIEKIFKVVYPVDDHHFKVILHYGT LVIDGKTPNMIDYFGRPYEGIAVFDGRKITVTGTLWNGNKIIDERLILPD GHLLFRVTINGVTGWRLCERILA
A.07	MVFTLEDFVGDWRQTAGYNLDQVLEQGGVSSLFQNLGVSVTPIQRIVL SGENGLKIDIHVIIPYEGLSGDQMGQIEKIFKVVYPVDDHHFKVILHYGT LVIDGVTPNMIDYFGRPYEGIAVFDGKKITVTGTLWNGNKIIDERLINPD GSLLFRVTINGVTGWRLCERILASEHHH
A.08	MHMHHMVFTLEDFVGDWRQTAGYNLDQVLEQGGVSSLFQNLGVSVT PIQRIVLSGENGLKIDIHVIIPYEGLSGDQMGQIEKIFKVVYPVDDHHFKV ILHYGTLVIDGVTPNMIDYFGRPYEGIAVFDGKKITVTGTLWNGNKIIDE RLINPDGSLLFRVTINGVTGWRLCERILA
A.09	MTHTLEDFVGDWRQTAGYNLDQVLEQGGVSSLFQNLGVSVTPIQRIVL SGENGLKIDIHVIIPYEGLSGDQMGQIEKIFKVVYPVDDHHFKVILHYGT LVIDGVTPNMIDYFGRPYEGIAVFDGKKITVTGTLWNGNKIIDERLILED GSLLFRVTINGVTGWRLCERILA
A.10	MVFTLEDFVGDWRQTAGYNLPQVLEQDGVSSLFQNLGVSVTPIQRIVL SGPNGLKIDIHVIIPKEGLSGDQMGQIEKIFKVVYPVDDHHFKVILHYGT LVIDGVTPNMIDYFGRPYEGIAVFDGKKITVTGTLWNGNKIIDERLINPD GSLLFRVTINGVTGWRLCERILA

B.01 (WT)	MVFTLEDFVGDWRQTAGYNLDQVLEQGGVSSLFQNLGVSVTPIQRIVL SGENGLKIDIHVIIPYEGLSGDQMGQIEKIFKVVYPVDDHHFKVILHYGT LVIDGVTPNMIDYFGRPYEGIAVFDGKKITVTGTLWNGNKIIDERLINPD GSLLFRVTINGVTGWRLCERILA
B.02	MTHTLEDFVGDWRQTAGYNLDQVLEQGGVSSLFQNLGVSVTPIQRIVL HGENGLKIDIAVIIPYEGLSGDQMGQIEKIFKVVYPVDDHHFRVILHYGT LVIDGKTPNMIDYFGRPYEGIAVFDGRKITVTGTLWNGNKIIDERLILED GHLLFRVTINGVTGWRLCERILA
B.03	MTHTLEDFVGDWRQTAGYNLDQVLEQGGVSSLFQNLGVSVTPIQRIVL HGENGLKIDIAVIIPYEGLSGDQMGQIEKIFKVVYPVDDHHFRVILHYGT LVIDGKTPNMIDYFGRPYEGIAVFDGRKITVTGTLWNGNKIIDERLINED GHLLFRVTINGVTGWRLCERILA
B.04	MTHTLEDFVGDWRQTAGYNLDQVLEQGGVSSLFQNLGVSVTPIQRIVL SGENGLKIDIAVIIPYEGLSGDQMGQIEKIFKVVYPVDDHHFRVILHYGT LVIDGVTPNMIDYFGRPYEGIAVFDGKKITVTGTLWNGNKIIDERLILED GSLLFRVTINGVTGWRLCERILA
B.05	MTHTLEDFVGDWRQTAGYNLDQVLEQGGVSSLFQNLGVSVTPIQRIVL HGENGLKIDIHVIIPYEGLSGDQMGQIEKIFKVVYPVDDHHFKVILHYGT LVIDGKTPNMIDYFGRPYEGIAVFDGRKITVTGTLWNGNKIIDERLILED GHLLFRVTINGVTGWRLCERILA
B.06	MTFTLEDFVGDWRQTAGYNLDQVLEQGGVSSLFQNLGVSVTPIQRIVL SGENGLKIDIAVIIPYEGLSGDQMGQIEKIFKVVYPVDDHHFRVILHYGT LVIDGVTPNMIDYFGRPYEGIAVFDGKKITVTGTLWNGNKIIDERLILPD GSLLFRVTINGVTGWRLCERILA
B.07	MVFTLEDFVGDWRQTAGYNLDQVLEQGGVSSLFQNLGVSVTPIQRIVL HGENGLKIDIAVIIPYEGLSGDQMGQIEKIFKVVYPVDDHHFRVILHYGT LVIDGKTPNMIDYFGRPYEGIAVFDGRKITVTGTLWNGNKIIDERLILPD GHLLFRVTINGVTGWRLCERILA
B.08	MTHTLEDFVGDWRQTAGYNLDQVLEQGGVSSLFQNLGVSVTPIQRIVL SGENGLKIDIAVIIPYEGLSGDQMGQIEKIFKVVYPVDDHHFRVILHYGT LVIDGVTPNMIDYFGRPYEGIAVFDGKKITVTGTLWNGNKIIDERLILPD GSLLFRVTINGVTGWRLCERILA
B.09	MHHTLEDFVGDWRQTAGYNLDQVLEQGGVSSLFQNLGVSVTPIQRIVL HGENGLKIDIAVIIPYEGLSGDQMGQIEKIFKVVYPVDDHHFRVILHYGT LVIDGKTPNMIDYFGRPYEGIAVFDGQKITVTGTLWNGNKIIDERLILPD GHLLFRVTINGVTGWRLCERILA
B.10	MTHTLEDFVGDWRQTAGYNLDQVLEQGGVSSLFQNLGVSVTPIQRIVL HGENGLKIDIAVIIPYEGLSGDQMGQIEKIFKVVYPVDDHHFRVILHYGT LVIDGKTPNMIDYFGRPYEGIAVFDGRKITVTGTLWNGNKIIDERLILPD GHLLFRVTINGVTGWRLCERILA

Table S8. Mutational details.

Sequence ID	Mutations from wild-type	Mutation Rationale	Positions Allowed to Mutate	Reference Structure for BayesDesign
A.01	H59A, K91R	Rational Design	None	None
A.02	V40T, H59A, K91R	Rational Design	None	None
A.03	H59A, K91A	Alanine Substitutions	None	None
A.04	V40A, H59A, K91A	Alanine Substitutions	None	None
A.05	I62L, P63S, Y64K, E65D, M72D, G73Q, Q74E, E76K, I78V, V81H, V82I	BayesDesign Helix Regions	62-65, 71-84	7SNT
A.06	S49H, V104K, K125R, N146L, S150H	BayesDesign Loop Regions	49-52, 101-106, 121-125, 145-150	7SNT
A.07	A172S, A173E, A174H, A175H, A176H	BayesDesign C-Terminus	172-176	7SNT+AAAAA
A.08	A-4M, A-3H, A-2M, A-1H, A0H	BayesDesign N-Terminus	-4-0	AAAAA+7SNT
A.09	V2T, F3H, N146L, P147E	BayesDesign on Shortest Path Method N-Terminus Correlation	2-6, 146-147	7SNT
A.10	D21P, G27D, E51P, Y64K	BayesDesign on Shortest Path Method Minimal Correlation	7, 8, 21, 27, 51, 63-64, 85-86, 99, 106, 120, 128, 145, 149	7SNT
B.01	Wild-type	None	None	None
B.02	V2T, F3H, S49H, H59A, K91R, V104K, K125R, N146L, P147E, S150H	Combinatorial Design	None	None
B.03	V2T, F3H, H59A, K91R, N146L, P147E	Combinatorial Design	None	None
B.04	V2T, F3H, S49H, V104K, K125R,	Combinatorial Design	None	None

	N146L, P147E, S150H			
B.05	H59A, K91R, N146L	Combinatorial Design	None	None
B.06	V2T, F3H, S49H, H59A, K91R, V104K, K125R, P147E, S150H	Combinatorial Design	None	None
B.07	S49H, H59A, K91R, V104K, K125R, N146L, S150H	BayesDesign In Combinatorial Space	49, 104, 125, 146, 150	7SNT + H59A, K91R (wild-type + A.01)
B.08	V2T, F3H, H59A, K91R, N146L	BayesDesign In Combinatorial Space	2-3, 146-147	7SNT + H59A, K91R (wild-type + A.01)
B.09	V2H, F3H, S49H, H59A, K91R, V104K, K125Q, N146L, S150H	BayesDesign In Combinatorial Space	2-6, 49-52, 101-106, 121-125, 145-150	7SNT + H59A, K91R (wild-type + A.01)
B.10	V2T, F3H, S49H, H59A, K91R, V104K, K125R, N146L, S150H	BayesDesign In Combinatorial Space	2-3, 146-147	7SNT + S49H, H59A, K91R, V104K, K125R, N146L, S150H (wild-type + A.01 + A.06)

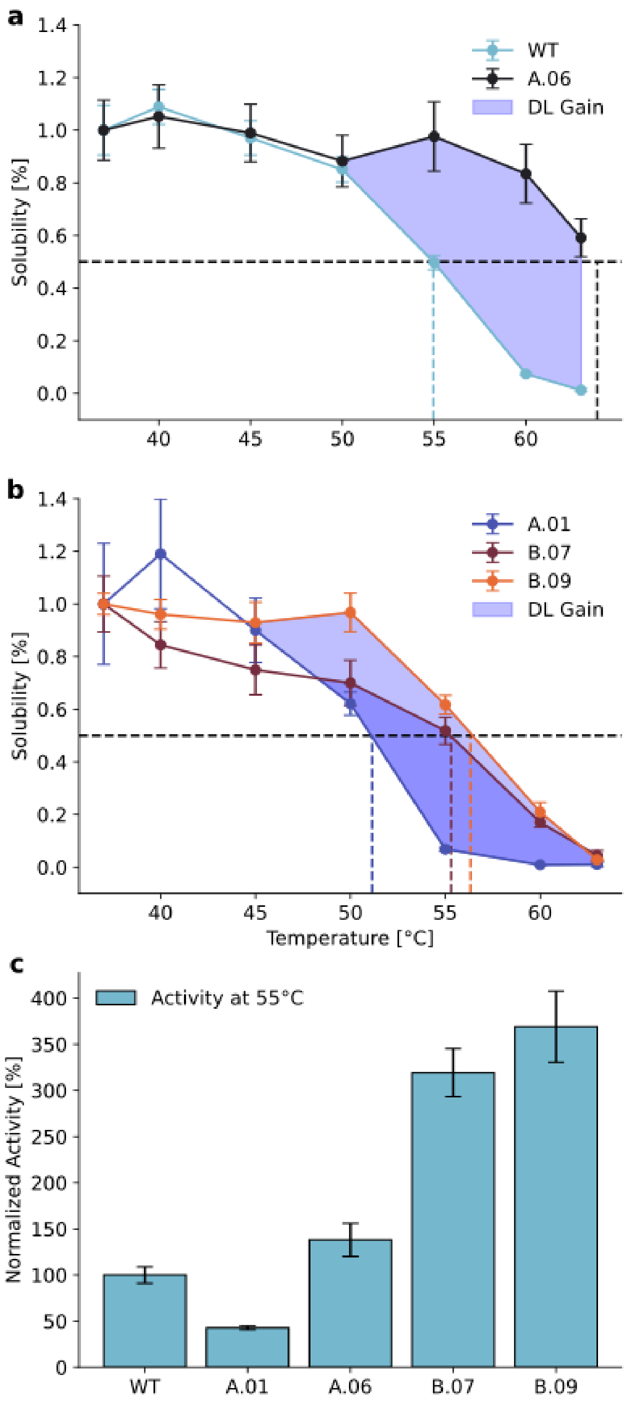

Figure S7. Solubility and post-freeze-thaw activity of promising NLuc variants. *a) Solubility of variant A.06 compared with wild-type. Blue shaded area indicates solubility gains achieved through our deep learning (DL) protocol. b) Solubility comparison of A.01-based variants. Blue shading highlights improvements attributable to DL methodology. Dashed lines identify* T_{50} *values. c) Activity of NLuc variants at elevated temperature after freeze-thaw, normalized to wild-type luminescence.*

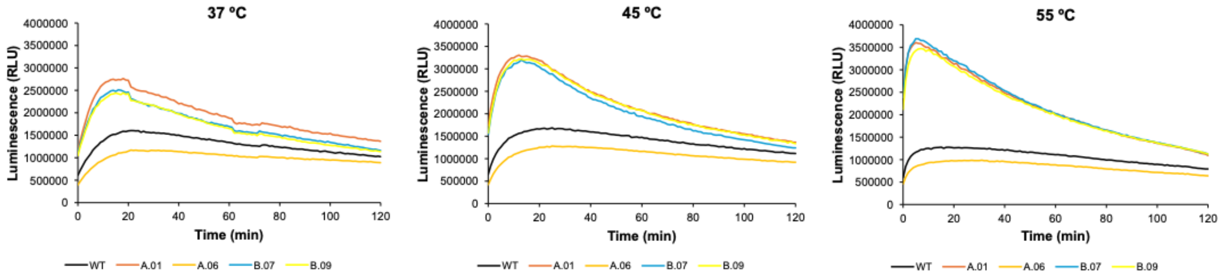

Figure S8. Time-course profiles. *The activity of the selected variants was measured for 2 hours under varying temperature conditions (37, 45, and 55 °C) at fixed enzyme (5 nM) and substrate (100 µM) concentrations to determine their time-course profiles.*

Supplementary Methods & Results

Freeze-thaw Method

Protein sequences from Figure S7c were heat treated at 55 °C for 15 minutes, followed by a subsequent freeze/thaw cycle for one day, and then measured for activity at 55 °C after 30 seconds of shaking by the plate reader and waiting for an additional 150 seconds (totaling 3 minutes after substrate addition). Proteins were diluted to an equal concentration in PBS buffer before freeze/thaw cycle. The activity of each variant was normalized to the WT protein. Activity assay conditions were identical to those performed in the initial screening campaign.

Freeze-thaw Results

Temperature-dependent activity assays revealed striking differences in the thermal performance profiles of NLuc variants after freeze-thaw. At 55 °C, variant A.01 exhibited significantly diminished activity (42.7 ± 1.9% of wild-type), consistent with its reduced thermostability and accelerated denaturation kinetics (Figure 5). In contrast, the stabilized A.06 variant demonstrated enhanced catalytic efficiency (138.1 ± 18.0 % relative to wild-type) under these conditions. The engineered variants B.07 and B.09 displayed improved thermal stability over A.01, achieving 319.3 ± 26.0 % and 369.0 ± 38.6 % of wild-type activity, respectively.

Time-Course Methods

A 1 mM FMZ solution was prepared by dissolving solid FMZ into Nano-Glo® Luciferase Assay Buffer. 90 µL of 5.55 nM enzyme solutions were added to a 96 well plate and preheated to 37, 45, or 55 °C. 10 µL of FMZ solution were added to the enzyme solutions and activity was measured in a Biotek SynergyMx plate reader at 37, 45, or 55 °C. A luminescence measurement was then taken every minute for 2 hours starting 15 seconds after substrate addition which includes a 10-second shaking period by the plate reader. Each measurement was run in triplicate. It should be noted that the initial time points (t=0) for the 55 °C data were used in Figure 6b as the 100 µM data points for kinetic parameter analysis.

Time-Course Results

The time course data shows that A.01 exhibited the highest activity at 37 °C. However, when heated, both B.07 and B.09 had activities that were nearly identical to A.01, demonstrating that the stability mutations overcome the stability-activity tradeoff. Additionally, even after 2 hours, variants A.01, B.07, and B.09 exhibited higher activity than both wild-type and A.06 variants. Variant A.06 displays a very similar time course profile as wild-type.

References

1. Abramson, J., et al., *Accurate structure prediction of biomolecular interactions with AlphaFold 3*. Nature, 2024. **630**(8016): p. 493–500.
2. Nemergut, M., et al., *Illuminating the mechanism and allosteric behavior of NanoLuc luciferase.* Nature Communications, 2023. **14**(1): p. 7864.
3. Chen, J.Y.-H., et al., *De novo luciferases enable multiplexed bioluminescence imaging.* Chem, 2025. **11**(3): p. 102346.
